# TM-Loop: Transformer multi-omics hierarchical detection of chromatin loop

**DOI:** 10.1038/s41598-026-56418-4

**Published:** 2026-06-11

**Authors:** Haixia Zhai, Hao Yang, Zhanwei Hou, Xiaoyan Liu, Junfeng Wang

**Affiliations:** School of Software, Henan Polytechnic University, Jiaozuo, 454003 China

**Keywords:** Chromatin loop, Hi-C, Multiomics data, Deep learning, Transformer, Threshold, Cancer, Computational biology and bioinformatics

## Abstract

Chromatin loops form the hierarchical 3D genome architecture and act as critical regulatory hubs, bringing distant cis-elements together to precisely control target gene transcription. Disruption of these spatial interactions is closely linked to human diseases including cancer. Hi-C provides genome-wide interaction maps, yet robust identification of chromatin loops remains a major bottleneck in 3D genomics, especially for contact matrices with low signal-to-noise ratio and high sparsity. We propose TM-Loop, a framework for accurate chromatin loop detection that integrates multi-omics data, Transformer deep learning, and hierarchical multi-scale clustering. Using 10 kb Hi-C matrices as core data, it combines ATAC-seq and CTCF ChIP-seq signals to build a weighted feature system and reduce sample imbalance. The Transformer’s multi-head attention captures global and local feature dependencies, while dual-threshold filtering and anchor-guided clustering effectively remove false signals and lower false discovery rate. Source code: https://github.com/yimuhuashui/TM-Loop. Experiments show TM-Loop outperforms existing methods in APA, protein enrichment, and 3D structural consistency, providing a new tool for high-precision genome-wide chromatin loop analysis.

**Supplementary Information:**

The online version contains supplementary material available at 10.1038/s41598-026-56418-4.

## Introduction

 As the core functional unit of the eukaryotic three-dimensional genome architecture, the chromatin loop can anchor and connect genome segments (such as enhancers and promoters) with long linear distance but closely related functions in space, and then accurately regulate the temporal and spatial specificity of gene expression^1^. This special loop topology is mainly formed by the mediation of CTCF-cohesin protein complex. Its dynamic reconstruction process is closely related to cell differentiation, fate decision and physiological function maintenance, and structural abnormalities often become an important cause of disease^2^. These abnormalities are frequently driven by genomic insertions and deletions, whose accurate detection is critical for mechanistic studies^3,4^. Previous studies have confirmed that the point mutation of SBE2 enhancer will destroy its regulatory loop with SHH gene, and eventually lead to forebrain malformation syndrome^5^; Enhancer hijacking near the MYC locus can induce abnormal chromatin loops, drive the continuous activation of oncogenes and promote the occurrence and development of tumors^6^. These findings fully show that the analysis of the spatial organization mode of chromatin loop is the key link to reveal the essence of life regulation and the pathogenesis of disease. In fact, the three-dimensional folding of chromatin will form a hierarchical structural system, covering multiple levels such as A/B compartments, topological Association domains (TADs) and chromatin loops^7^. Most of the research methods for this complex system are derived from chromosome conformation capture technology (3 C)^8^, and continue to improve and innovate with the technology iteration.

The emergence of high-throughput chromosome conformation capture technology (Hi-C)^9,10^ provides a revolutionary tool for genome-wide chromatin interaction research. This technology converts the chromatin spatial interaction information into a quantifiable contact probability matrix through a standardized experimental process. After ten years of development, its detection flux has jumped from a million level interaction pairs to a hundred billion level, and the genome resolution has also achieved a leap from 1 MB to 1 KB^11^. However, the massive data brought about by technological progress has also given birth to a new computational challenge - how to accurately identify the biological significance of chromatin loop structure from the contact matrix filled with systematic noise and technical deviation. This problem essentially belongs to the category of high-dimensional sparse signal detection: in the 10 KB resolution Hi-C matrix, the effective signal corresponding to the real chromatin loop only accounts for about 0.01% of the total pixels. Combined with the influence of insufficient sequencing depth, enzyme digestion preference, PCR amplification bias and other factors, the problem of signal-to-noise ratio imbalance of data is further aggravated, which sets many obstacles to the accurate identification of chromatin loop.

At present, the computational methodology of chromatin loop prediction has completed the paradigm transformation from traditional statistical model to machine learning technology, and the methods of different technical routes have their own advantages and disadvantages. Deep learning technology has shown wide application value in the field of genomics related analysis. It not only plays an important role in chromatin ring recognition, but also has made breakthroughs in related directions such as topology related domains (TADS) recognition, haplotype assembly and genome variation detection. For example, MAMnet realizes accurate detection of insertion and deletion variation based on long reading and long data and deep learning methods^12^, DeepHapNet optimizes haplotype assembly effect with retnet and deep spectral clustering technology^13^, deepTAD combines convolutional neural network and transformer model to improve TADS recognition performance^14^. The supervised learning method driven by deep learning shows significant advantages in the task of chromatin loop recognition with its powerful feature mining ability: peakachu^15^ took the lead in applying the random forest algorithm to contact matrix classification, opening the precedent of machine learning to predict chromatin loops; Dloopcaller^16^ integrated Hi-C data and chromatin accessibility information to build a multi data joint prediction model; GILoop^17^ designed a double branch neural network to synchronously learn pixel level features and topological correlation features to improve the prediction accuracy; Be-1DCNN^18^ enhances the robustness of the model by integrating multiple one-dimensional convolutional networks; In contrast, the early unsupervised methods were mostly developed based on the principle of local enrichment statistics. Although they can effectively identify strong signal loops, they are not sensitive to weak interaction structures: HiCCUPS innovatively used multi-scale Poisson test to compare the contact abundances of target pixels and four types of background areas to screen significant interactions; Fit-Hi-C^19^ combined with the characteristics of data deviation to build a statistical model to evaluate the significance of medium-range chromosome interactions; SIP^20^ uses high-intensity interaction sites as clues to predict the chromatin loop, and filters false positives through the region maximization algorithm; Mustache^21^ introduced the scale space theory of computer vision and identified the speckled enrichment feature with the help of Gaussian difference filter; Chromosight^22^ uses the matrix balance normalization to correct the technical deviation and extract the local peak to determine the loop anchor; HiCExplorer^23^ performs background-corrected local enrichment analysis combined with statistical testing to identify significant chromatin loops from normalized Hi-C contact matrices. In addition, CD-loop^24^ combined with contact density matrix and dynamic threshold to screen potential loop structures, Dconnloop^25^ identified functional loops based on graph theory connectivity analysis, CGloop^26^ fused genome context features and convolutional network capture loop features., and YOLOOP^27^ adopted a lightweight detection architecture to efficiently mine local interaction patterns for rapid chromatin loop recognition. It is worth noting that the existing methods still have common shortcomings, such as single feature extraction dimension, high false positive rate, and poor adaptability to low depth sequencing data. The unique advantages of deep learning technology in mining hidden features of complex data provide a feasible path to break through these bottlenecks.

Here, we propose TM-Loop, a framework for accurate detection of chromatin loops that integrates multi omics data integration, transformer deep learning architecture and hierarchical multi-scale clustering optimization. The framework takes the 10 KB resolution Hi-C contact matrix as the core data, innovatively integrates ATAC-seq chromatin accessibility data and CTCF ChIP-seq protein binding data, and constructs a differentiated weighted feature system of “Hi-C core structure features + multi omics function enhancement features”, so as to alleviate the imbalance between positive and negative samples from the data source; Relying on the multi head attention mechanism of transformer architecture, we can deeply mine the global association and local dependence between features, and strengthen the feature saliency of key anchor areas; Combined with the hierarchical multi-scale clustering strategy of double threshold step-by-step screening and anchor guidance, the high-efficiency filtering of false interactive signals is realized, and the false positive rate of prediction results is significantly reduced. Experimental verification shows that TM-Loop is superior to the existing mainstream methods in core evaluation indicators such as APA analysis^28,29^, binding protein enrichment^30–32^ and three-dimensional structure consistency, and provides a new technical tool for genome-wide accurate analysis of chromatin loops.

## Methods

**Fig. 1:**
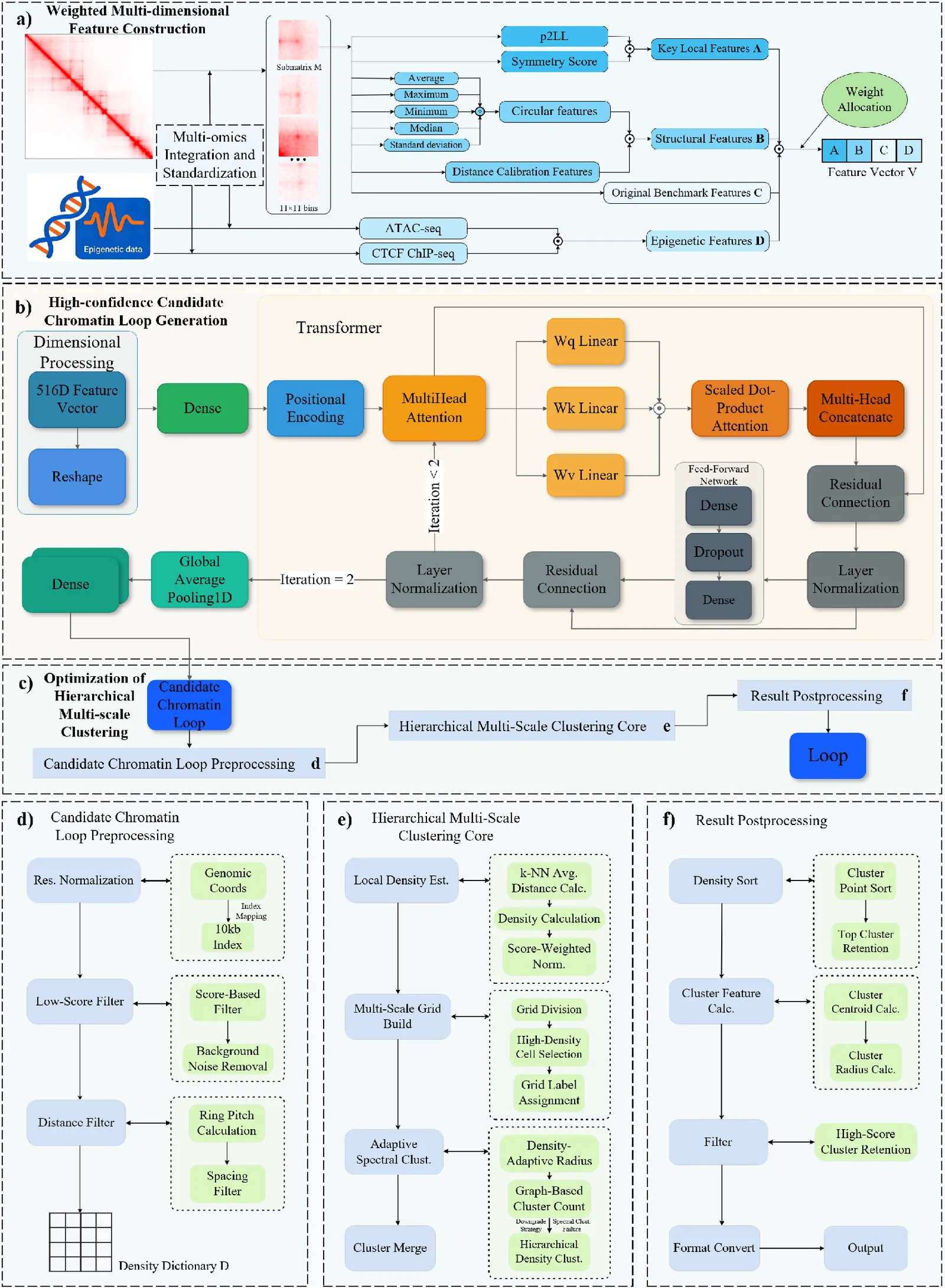
TM-Loop workflow: (a) Multi-omics Integration and Standardization. (b) Weighted Multi-dimensional Feature Construction. (c) High-confidence Candidate Chromatin Loop Generation. (d) Optimization of Hierarchical Multi-scale Clustering.

TM-Loop uses a 10KB resolution Hi-C contact matrix and integrates ATAC-seq and CTCF ChIP-seq three-dimensional genome modification data to achieve high-precision and low false positive chromatin loop detection. The process includes four core stages: (I) Multi-omics Integration and Standardization: firstly, the method integrates Hi-C, ATAC-seq and CTCF ChIP-seq heterogeneous data, performs KR normalization and sparse processing on Hi-C data, windowed extraction and missing value filling on epigenetic data, and constructs a balanced positive and negative sample set to alleviate the problem of category imbalance; (II) Weighted Multi-dimensional Feature Construction: for each preprocessed genome window region, the method extracts multi-dimensional features such as structural features and epigenetic modification features, and realizes feature fusion through differential weighting strategy, which is transformed into a unified 516 dimensional feature vector, playing the role of multi data complementary verification to eliminate single data noise; (III) High-confidence Candidate Chromatin Loop Generation: this method uses the pre training deep learning framework, outputs the prediction probability based on the multi-dimensional weighted feature vector, and generates high confidence candidate chromatin loops through the double threshold screening strategy, which significantly reduces the false positive rate; (IV) Optimization of Hierarchical Multi-scale Clustering: finally, the method uses the adaptive density hierarchical multi-scale clustering algorithm to optimize the detection results, eliminate redundant candidate loops, and output high-quality, non redundant final chromatin loop data sets. The workflow of TM loop is shown in Fig. 1.

### Multi-omics integration and standardization

This method takes the multi-dimensional omics data as the input, eliminates the technical deviation and dimensional difference through the unified pretreatment process, and constructs the balanced sample set, which lays the foundation for the subsequent feature extraction and prediction. The input data contains three types of core omics data: 10 kb resolution Hi-C contact matrix (.cool/.hic format); ATAC-seq data (BigWig format) reflects the accessibility of chromatin region; CTCF ChIP-seq data (BigWig format) were used to record the binding sites of key anchor proteins in the chromatin loop.

According to the characteristics of heterogeneous data, a targeted preprocessing process was designed: KR normalization was used for Hi-C data to eliminate technical deviations such as uneven sequencing depth and enzyme digestion preference, and non-zero interaction sites were screened through sparse processing to retain high confidence spatial interaction information; For ATAC-seq and CTCF ChIP-seq data, an 11 × 11 window (corresponding to 110 kb genome interval) was constructed with each Hi-C interaction site as the center, and the signal values in the window were extracted. Missing values in the signals were filled by zero filling.

During the construction of the sample set, the positive samples were determined based on CTCF ChIA-PET and H3K27ac HiChIP gold standard data, and the negative samples were generated by kernel density estimation (KDE) combined with uniform sampling. The number of negative samples was strictly controlled not to exceed three times of the positive samples to alleviate the imbalance between positive and negative samples. The formula is as follows:

1 $$\:\begin{array}{c}{neg}_{num}=min\left({neg}_{candidates},3\times\:{pos}_{num}\right)\end{array}$$

Where *neg*_*candidates*_ is the total number of candidate negative samples and *pos*_*num*_ is the number of positive samples. At the same time, carry out strict boundary verification: ensure that each interaction site in the Hi-C matrix is at least 5 bin away from the matrix boundary to avoid incomplete windows. If the boundary is too close, the anchor position will be automatically adjusted, and if it cannot be adjusted, the site will be eliminated; Ensure that the length of the signal sequence after windowed extraction of epigenetic data is unified as 110 kb. If it is insufficient, zero is added, and if it exceeds, it is truncated. The formula is as follows:

2 $$\:\begin{array}{c}{signal}_{norm}=\left\{\begin{array}{c}signal\\\:pad\left(signal,110000\right)\\\:signal\left[0:110000\right]\end{array}\begin{array}{c}if\:len\left(signal\right)=11000\\\:if\:len\left(signal\right)<11000\\\:if\:len\left(signal\right)>11000\end{array}\right.\end{array}$$

### Weighted multi-dimensional feature construction

The accurate identification of chromatin loop needs to take the spatial interaction characteristics reflected by Hi-C data as the core, and integrate the functional regulation information provided by multi omics data to form a feature system of “core structure+function enhancement”. Based on the framework of Hi-C structural features, this study takes the multi omics features as the key enhancement module, through fine extraction and differential fusion, comprehensively captures the spatial conformation and functional regulation characteristics of the chromatin loop, and highlights the core value of multi omics data in eliminating noise and supplementing dimensions, as shown in Fig. 1(a).

(1) Core framework: Hi-C structure feature extraction.

Hi-C data is the core basis for chromatin loop detection. Its structural features directly reflect the spatial interaction mode of chromatin, and build the basic framework of the feature system, covering five key dimensions:

① P2LL.

Quantify the enrichment significance of local interaction signals, and calculate the intensity ratio between the central pixel and the edge background region:

3 $$\:\begin{array}{c}{f}_{p2LL}=\frac{{v}_{c}}{{\mu\:}_{bg}+{10}^{-7}}\end{array}$$

Where *V*_*C*_ is the interaction intensity of the central pixel of the submatrix, *µ*_*bg*_ is the average intensity of the edge 1/5 region, and the denominator plus 10^− 7^ avoids zero error, forming a one-dimensional key discriminant feature.

② Symmetry score.

The real chromatin loops are approximately symmetrically distributed in the Hi-C matrix. The symmetry is quantified by the difference between the submatrix and its transpose. The formula is as follows:

4 $$\:\begin{array}{c}{f}_{sym}=1-\frac{{\left|\left|{SubM}_{ij}-{{SubM}_{ij}}^{T}\right|\right|}_{F}}{{\left|\left|{SubM}_{ij}\right|\right|}_{F}+{10}^{-7}}\end{array}$$

Where, *||·||*_*F*_ represents the Frobenius norm of the matrix, and the closer the score is to 1, the higher the symmetry is, which is an important 1-dimensional auxiliary feature.

③ Circular features.

Quantify the radial spatial distribution of chromatin loops, and extract the statistical features of concentric rings layer by layer from the center of the extracted sub matrix window. For radius *r* = 0,1., 5 ring region: *r* = 0 corresponds to the center point, *r* > 0 samples are taken along 8*r* uniform angles, and the mean *µ*_*r*_, maximum *max*_*r*_, minimum *min*_*r*_, median *median*_*r*_, and standard deviation *σ*_*r*_ of each ring region are calculated to form a five-dimensional sub feature. After the sub features of all radii are spliced, a 30 dimensional circular eigenvector *f*_*ring*_ is obtained to characterize the spatial structure details of the chromatin loop.

④ Distance calibration features.

The inherent attenuation effect of genome distance on Hi-C interaction frequency will mask the real chromatin loop signal, and the signal correction is based on the power law relationship:

5 $$\:\begin{array}{c}genomi{c}_{dist}=res\times\:\frac{\sqrt{{max}\left({\left(i-w\right)}^{2}+{\left(j-w\right)}^{2}\right),0.1)}}{1000}\end{array}$$

Where, 10,000 is the genome resolution (BP), *w* is the window radius, *(i*,* j)* is the pixel coordinates in the 11 × 11 window, divided by 1000 to convert the unit to kb, *max*(…, 0.1) to avoid the center pixel distance being zero.

Based on the empirical decay law of mammalian cell chromatin contact probability, a distance correction matrix was constructed:

6 $$\:\begin{array}{c}{dis{t}_{matrix}}_{i,j}={{max}\left(genomi{c}_{dist},10\right)}^{0.75}\end{array}$$

The index of 0.75 is derived from the empirical decay coefficient of mammalian cell chromatin contact probability. Finally, the real interaction signal is enhanced by calibration matrix:

7 $$\:\begin{array}{c}{correcte{d}_{SubM}}_{i,j}={SubM}_{i,j}\times\:{dis{t}_{matrix}}_{i,j}\end{array}$$

The corrected matrix is flattened to form a 121 dimensional feature vector *f*_*dist*_, which is the core dimension of the feature system.

⑤ Original benchmark features.

To retain the original signal reference, the original signals of Hi-C submatrix, ATAC-seq and CTCF ChIP-seq are flattened directly to form the 121 dimensional original datum feature vector *f*_*raw*_.

(2) Enhancement module: multi omics feature depth extraction.

As the key enhancement of core structural features, multiomics features supplement the discrimination information of chromatin loop from the functional regulation level, realize the dual characterization of “spatial structure + functional activity”, and highlight the synergy with Hi-C structural features in the extraction process:

① Feature extraction logic.

According to the 11 × 11 window (110 kb genome interval) of each Hi-C interaction site, ATAC-seq (chromatin accessibility) and CTCF ChIP-seq (anchored protein binding) data were synchronously extracted. The authenticity of the chromatin loop was verified from the “chromatin open state” and “molecular mechanism of loop formation”, which were complementary to the spatial structure characteristics of Hi-C.

② Refined processing flow.

Firstly, the original multi omics signal is preprocessed: missing values were filled by zero filling to ensure signal stability. In order to reduce data redundancy and retain key information, kernel principal component analysis (KPCA) is used to reduce dimension:

8 $$\:\begin{array}{c}kpc{a}_{feat}=KPCA\left(signal,{n}_{components}=50,kernel={\prime\:}rb{f}^{{\prime\:}},\gamma\:=0.06\right)\end{array}$$

Among them, *γ* = 0.01 is the optimal bandwidth parameter of RBF kernel function, which is determined through empirical verification.

After dimensionality reduction, the synergetic characteristics of the interaction strength between multi omics signals and Hi-C were calculated:

9 $$\:\begin{array}{c}{synergy}_{i,j}=corr\left({kpc{a}_{multiomics}}_{i},{Hi{C}_{intensity}}_{j}\right)\end{array}$$

Among them, *corr* is Pearson correlation coefficient, which quantifies the synergistic relationship between epigenetic signals and spatial interaction. Min-Max normalization of collaborative characteristic matrix:

10 $$\:\begin{array}{c}{synergy}_{norm,i,j}=\frac{{synergy}_{i,j}-\mathrm{min}\left(synergy\right)}{\mathrm{max}\left(synergy\right)-\mathrm{min}\left(synergy\right)+{10}^{-7}}\end{array}$$

Finally, the normalized matrix is flattened to form 121 dimensional ATAC-seq eigenvector *f*_*atac*_ and 121 dimensional CTCF ChIP-seq eigenvector *f*_*ctcf*,_ respectively.

(3) Feature fusion and weighted optimization.

The differentiated weighting strategy is designed based on the principle of “giving priority to the core structure, supplemented by multi omics enhancement”:

1) standardize the Z-score according to the feature groups (mean subtraction and standard deviation Division) to eliminate the dimensional differences between different feature groups;

2) give the highest weight to the key local features of Hi-C: the center background ratio *f*_*p2ll*_ and symmetry *f*_*sym*_ weight 2.0;

3) give the second highest weight to the Hi-C structure features: the distance correction feature *f*_*dist*_ and the circular feature *f*_*ring*_ weight of 1.5;

4) give the multi omics epigenetic features moderate weight: the weight of ATAC-seq feature *f*_*atac*_ and CTCF ChIP-seq feature *f*_*ctcf*_ is 1.2, highlighting its function verification value;

5) The fraw weight of the original benchmark features are 1.0 as the basic reference

Among them, the distribution of feature weight strictly matches the biological contribution of each feature to the discrimination of chromatin loop - the core discriminant factor (center background ratio, symmetry) is given the highest weight of 2.0, the global structural support (distance correction, ring feature) is given the second highest weight of 1.5, the functional verification supplement (ATAC, CTCF multi omics feature) is given the medium weight of 1.2, and the original Hi-C feature of the reference is given the weight of 1.0, which fully conforms to the nature of the spatial conformation and functional regulation of chromatin loop. Finally, all weighted features are spliced in the order of “Key local features - structural features - multiomics enhanced features- original datum features " to form a 516 dimensional final feature vector *V*_*ij*_:

11 $$\:\begin{array}{c}{V}_{ij}\:=\:\left[{{f}_{p2LL};{\:f}_{sym};\:{f}_{ring};\:f}_{dist};\:{f}_{atac};\:{f}_{ctcf};\:{f}_{raw};\right]\end{array}$$

This vector not only retains the spatial core information of Hi-C data, but also complements the functional regulation dimension through multiomics features, so as to achieve a comprehensive and accurate characterization of chromatin loops.

### High-confidence candidate chromatin loop generation

Based on the constructed 516 dimensional multi-dimensional weighted feature vector, this study uses the encoder only transformer architecture (only including encoder module, not including decoder) as the core prediction model. Through the synergy of position coding, multi head attention, feedforward network and residual connection, the global association and local dependence between features are deeply mined, and combined with the double threshold screening strategy, the candidate chromatin loop with low noise and high confidence is generated. This prediction process gives full play to the advantages of transformer in processing long sequence data to achieve accurate modeling and discrimination of chromatin loop characteristics. The overall architecture is shown in Fig. 1 (b).

(1) Feature preprocessing and sequence modeling.

First, the validity of the 516 dimensional feature vector *V*_*ij*_ is checked: low quality features with sparsity over 90%, dimensional anomalies and invalid features with infinite values are eliminated to ensure the reliability of the input data. To adapt to the processing characteristics of the transformer for sequence data, the eigenvector is regarded as a one-dimensional sequence with a length of 516 *X*=[*x*_*1*_, *x*_*2*_,…, *x*_*516*_], where *X*_*T*_ represents the t-dimensional eigenvalue.

In order to improve the efficiency of large-scale data processing, batch caching strategy is adopted to save the effective sequence characteristics as.Pkl files by chromosome and batch to avoid repeated calculation. The formula is as follows:

12 $$\:\begin{array}{c}cache\left(X,chrom,batc{h}_{id}\right)\to\:file:chro{m}_{batc{h}_{id}}.pkl\end{array}$$

The cached data can be directly used for subsequent prediction, significantly reducing the consumption of computing resources and processing time.

(2) Transformer prediction model core components.

Transformer model is composed of position coding layer, multi head attention layer, feedforward network layer, residual connection and layer normalization. Each component cooperates to realize the depth coding and classification prediction of features. The specific structure and formula are as follows:

① Feature extraction logic.

Due to the structural association between the hidden dimensions of feature sequence *X* (such as the transition from local features to global features), the location coding layer adds location information to each feature dimension to help the model capture the sequence order dependency. The formula is as follows:

13 $$\:\begin{array}{c}PE\left(t,2k\right)={sin}\left(\frac{t}{{10000}^{2k/{d}_{model}}}\right)\end{array}$$

14 $$\:\begin{array}{c}PE\left(t,2k+1\right)={cos}\left(\frac{t}{{10000}^{2k/{d}_{model}}}\right)\end{array}$$

Where, *t* is the feature dimension index (1 ≤ *t* ≤ 516), *d*_*model*_ = 516 is the model input dimension, and *k* is the dimension split index (0 ≤ *k* < *d*_*model*_/2). The sequence after adding the location code is:

15 $$\:\begin{array}{c}{X}_{pe}=X+PE\end{array}$$

② Multi-head attention layer.

The multi head attention layer captures the association information of different dimensions between features by computing multiple attention heads in parallel, and realizes the global feature interaction. First, project the position encoded sequence *X*_*pe*_ into the query, key, and value spaces:

16 $$\:\begin{array}{c}Q={X}_{pe}\cdot\:{W}_{Q}+{b}_{Q}\end{array}$$

17 $$\:\begin{array}{c}K={X}_{pe}\cdot\:{W}_{K}+{b}_{K}\end{array}$$

18 $$\:\begin{array}{c}V={X}_{pe}\cdot\:{W}_{V}+{b}_{V}\end{array}$$

Where *W*_*Q*_, *W*_*K*_, *W*_*V*_ ∈ $\:{R}^{{d}_{model}\times\:{d}_{model}}$ are the projection weight matrix, *b*_*Q*_, *b*_*K*_, *b*_*V*_ ∈$\:{R}^{{d}_{model}}$are the offset terms.

*Q*, *K*, *V* are divided into four heads, and each head dimension is *d*_*k*_=d_model_/*h* = 129:

19 $$\:\begin{array}{c}{Q}_{i}=Q\left[:,:,\left(i-1\right){d}_{k}:{id}_{k}\right],{K}_{i}=K\left[:,:,\left(i-1\right){d}_{k}:{id}_{k}\right],{V}_{i}=V\left[:,:,\left(i-1\right){d}_{k}:{id}_{k}\right]\end{array}$$

Each attention head calculates the zoom dot product attention:

20 $$\:\begin{array}{c}Attention\left({Q}_{i},{K}_{i},{V}_{i}\right)=softmax\left(\frac{{Q}_{i}\cdot\:{K}_{i}^{T}}{\sqrt{{d}_{k}}}\right)\cdot\:{V}_{i}\end{array}$$

Splice all attention head outputs and perform linear transformation to obtain the final output of multi head attention layer:

21 $$\:\begin{array}{c}MultiHead\left(Q,K,V\right)=Concat\left({Attention}_{1},\dots\:,{Attention}_{h}\right)\cdot\:{W}_{O}+{b}_{O}\end{array}$$

Where, *W*_*0*_ ∈ $\:{R}^{{d}_{model}\times\:{d}_{model}}$ is the output weight matrix, and *b*_*0*_ ∈ $\:{R}^{{d}_{model}}$ is the bias term.

③ Feed-forward network layer.

The feedforward network layer performs nonlinear transformation on the multi head attention output to strengthen the nonlinear correlation of local features, and uses two-layer fully connected network to realize:

22 $$\:\begin{array}{c}FFN\left(z\right)=\mathrm{max}\left(0,z\cdot\:{W}_{1}+{b}_{1}\right)\cdot\:{W}_{2}+{b}_{2}\end{array}$$

Where *W*_*1*_ ∈ $\:{R}^{{d}_{model}\times\:2048}\:$and *W*_*2*_ ∈ $\:{R}^{2048\times\:{d}_{model}}$ are the full connection layer weight matrix, *b*_*1*_ ∈ *R*^2048^ and *b*_*2*_ ∈ $\:{R}^{{d}_{model}}$ are the offset terms, and *max* (0, ⋅) is the relu activation function.

④ Residual connection & layer normalization.

To alleviate the gradient disappearance problem in deep network training, each transformer layer (multi head attention layer+feedforward network layer) introduces residual connection and layer normalization:

23 $$\:\begin{array}{c}{z}_{1}=LayerNorm\left({X}_{pe}+MultiHead\left(Q,K,V\right)\right)\end{array}$$

24 $$\:\begin{array}{c}{z}_{2}=LayerNorm\left({z}_{1}+FFN\left({z}_{1}\right)\right)\end{array}$$

Where, the layer normalization formula is:

25 $$\:\begin{array}{c}LayerNorm\left(z\right)=\frac{z-E\left[z\right]}{\sqrt{Var\left(z\right)+\epsilon}}\cdot\:\gamma\:+\beta\:\end{array}$$

*E*[*z*] and *Var*(*z*) are the mean and variance of the sequence respectively, *ε*=10^− 7^ to avoid zero error, *γ* and *β* are learnable parameters.

(3) Prediction and dual threshold filtering.

After being processed by the two-layer transformer encoder, the output sequence *Z*_*2*_ is globally averaged and pooled to obtain the global eigenvector:

26 $$\:\begin{array}{c}\stackrel{-}{z}=\frac{1}{{d}_{model}}\sum\:_{t=1}^{{d}_{model}}{z}_{2}\end{array}$$

Input the global eigenvector into the classification head, and output the prediction probability of the chromatin loop through the *softmax* function:

27 $$\:\begin{array}{c}{p}_{pred}=softmax\left(\stackrel{-}{z}\cdot\:{W}_{cls}+{b}_{cls}\right)\end{array}$$

Where *W*_*cls*_ ∈ $\:{R}^{{d}_{model}\times\:2}$ is the classification weight matrix, *b*_*cls*_ ∈ *R*^2^ is the classification bias term, and *p*_*pred*_^1^ is the probability that the site is a real chromatin loop.

The double threshold screening strategy is adopted to improve the quality of candidate loops: the first level screening takes 0.5 as the threshold, retains the candidate results with *p*_*pred*_^1^> 0.5, and initially filters the low confidence noise; The two-level screening raises the threshold and retains the high confidence candidate loops that meet the requirements.

The double threshold screening effectively balances the recall rate and accuracy rate, and minimizes the false positive rate while preserving the real chromatin loop.

### Optimization of hierarchical multi-scale clustering

Through the adaptive density clustering algorithm, the spatial proximity signals are aggregated and redundant results are eliminated to further improve the accuracy and consistency of chromatin loop detection, and finally high-quality data sets are output, as shown in Fig. 1C. First, the local density of each candidate loop is calculated based on the k-nearest neighbor algorithm to reflect its spatial aggregation characteristics:

28 $$\:\begin{array}{c}density\left(x\right)=\frac{1}{\frac{1}{k}{\sum\:}_{y\in\:k-NN\left(x\right)}euclidean\left(x,y\right)+{10}^{-10}}\end{array}$$

Where *k-NN* (*x*) represents the *k* nearest neighbors of the candidate loop *x*, *Euclidean* (*x*, *y*) is the Euclidean distance, and the greater the density value, the higher the degree of aggregation of the candidate loops in the region.

Hierarchical multi-scale clustering strategy is adopted: a three-level scale grid is constructed, candidate loops are allocated to grid cells of different scales, and high-density grid cells (density greater than the average) are selected as the core clustering region to reduce the clustering complexity; Adaptive spectral clustering is adopted in each grid cell, and the clustering radius is dynamically adjusted based on local density. The formula is as follows:

29 $$\:\begin{array}{c}radius\left(x\right)=\frac{2.0}{density\left(x\right)+{10}^{-10}}\end{array}$$

The number of clusters is automatically determined by graph connectivity analysis, and the candidate loops whose distance is less than the radius of both sides are clustered to form the initial cluster without manual intervention.

After clustering, post-processing optimization is carried out: small clusters with less than 3 samples are merged and fused with the nearest cluster with high confidence to avoid the loss of key signals; According to the average prediction probability of candidate loops in the cluster, the first 10,000 high confidence clusters are retained and the redundant results are eliminated; Select the candidate loop with the highest prediction probability in each cluster as the representative point, convert it into the physical coordinates of the genome, and write it into the bedpe format file, which contains key information such as chromosome, start position, end position, prediction probability, and is compatible with downstream genomics analysis tools^33^.

### Experiments

(1) Standard Set Construction (gold standard data set preprocessing and integration).

Before constructing positive and negative samples for the TM-Loop model, it is necessary to give priority to constructing a high-quality chromatin loop gold standard data set. As the core benchmark reference of supervised learning, the integrity and accuracy of the data set directly affect the training efficiency and generalization performance of the model. The core of the gold standard data comes from the experimentally verified CTCF ChIA-PET and H3K27ac HiChIP data. Both types of data are stored in BEDPE format - this is a standardized format for recording the interaction between chromatins in the field of genomics, which can synchronously retain the key information such as the chromosome number, start and end coordinates of a pair of interaction regions.

In order to realize the compatibility and adaptation between the gold standard data and the 10 kb resolution Hi-C data used in TM-Loop, a series of standardized preprocessing and integration operations need to be performed on the original BEDPE file. The specific process is as follows: first, automatically identify the number of file columns (compatible with the 6-column or 7-column standard BEDPE format), and avoid data reading exceptions caused by subtle differences in format; Subsequently, the chromosome names were standardized and corrected to supplement prefix marks for numbers without “chr” prefix to ensure consistency with hg19 genome annotation version, providing basic support for the mapping of subsequent genome coordinates to Hi-C matrix box coordinates.

On this premise, carry out the resolution normalization processing of genome coordinates: for the starting coordinates of the two pairs of interaction regions in the BEDPE file, perform the downward rounding alignment at a resolution of 10 kb (that is, divide the coordinate value by 10000 and then multiply by 10000), and set the ending coordinates as the starting coordinates plus 10,000 bp, so that each interaction region strictly matches the Hi-C matrix box size adopted by TM-Loop, eliminating the interference of coordinate resolution differences on subsequent feature extraction. Finally, the data preprocessed by a single file is internally de duplicated, and then the data set after the integration of multi-source validation data (CTCF ChIA-PET and H3K27ac HiChIP data) is re duplicated globally to eliminate the redundant interaction site records, so as to avoid the weight shift of model training caused by duplicate samples.

After the above pretreatment process, the integrated high-quality BEDPE format gold standard file is finally generated, which covers the real chromatin loop interaction information verified by experiments, and provides a unified and reliable reference data source for subsequent positive sample extraction and negative sample simulation construction.

(2) Training, verification and test set construction.

In order to weaken the influence of chromosome specific bias on the generalization performance of the model, TM-Loop uses the chromosome leave one cross validation method to complete the division of training set, validation set and test set based on gm12878 cell line chromosome 1–22 and X chromosome samples. The specific division logic is as follows: when model training is carried out for a target chromosome (such as chromosome 16), all samples corresponding to that chromosome are divided into independent test sets separately, and the remaining samples of chromosomes 1–15, 17–22 and X are used as data sources for the training set and verification set.

For the data sources of the training set and the validation set, the positive and negative samples are randomly divided into 10 equal parts, 9 of which are combined to form the training set, and the remaining one is used as the validation set, so that the ratio of the two is strictly maintained at 9:1, which not only ensures the sufficient amount of training data, but also ensures that the validation results are statistically representative. After the whole process of data processing, the training set and validation set contain 259,488 samples after deducting the independent test set samples. See table S2 and table S3 of supplementary document 1 for details such as the specific calculation time and the distribution number of chromosome samples in the process of sample generation.

(3) Positive and negative sample construction.

Based on the above data set division framework, TM-Loop uses the complementary advantages of dual source experimental data to build a sample set to achieve a comprehensive coverage of different types of chromatin loops. Among them, CTCF Chia PET data is outstanding in capturing remote chromatin interactions, which can effectively reflect the formation process of chromatin loop mediated by loop extrusion model^34^; H3k27ac hichip data is more focused on short-range interaction capture, which can accurately characterize the regulatory association between active enhancers and promoters^35^. The collaborative application of the two can make up for the short coverage of a single data type and broaden the detection range of chromatin loop.

① Positive sample construction.

Taking the integrated CTCF Chia pet and h3k27ac hichip gold standard data as the core, the real chromatin interaction sites verified by experiments were extracted, and these sites were mapped into the Hi-C contact matrix through the precise transformation of genome coordinates and corresponding resolution box coordinates. Then, the sites too close or too far away were eliminated by distance screening, and only the interaction regions with biological rationality were retained. For each effective site, the local contact matrix window is intercepted with it as the center, and the method of “multi-dimensional weighted feature construction” above is used to generate a vector that combines the Hi-C core structure features and multi omics enhanced features. At the same time, the samples with sparse window and insufficient significance of central interaction signal are strictly filtered to form a high-quality positive sample set, which provides a reliable positive reference for the model.

② Negative sample construction.

In order to reduce the interference of data bias on model training, negative samples are generated based on the distance distribution characteristics of positive samples. Firstly, based on the interaction distance of positive samples, the distance distribution model is constructed by kernel density estimation, and the short-range lower limit, long-range start and end thresholds are set to accurately define the short/long-range interaction. The generation of negative samples is divided into two parts: the short-range negative samples are sampled based on the kernel density estimation model to ensure that their distance distribution is highly consistent with the short-range characteristics of positive samples; The long-distance negative samples are randomly sampled according to the cumulative probability distribution within the preset long-distance range to supplement the negative samples of long-range interaction scenarios.

For each distance obtained by the two types of sampling, the site pairs that meet the distance are screened in the Hi-C contact matrix (the starting coordinate is r, and the ending coordinate is the sum of R and the corresponding distance), only the effective site pairs with contact strength greater than 0 are retained, and the site pairs that have been included in the positive sample set are eliminated to ensure that the positive and negative samples do not overlap. Finally, the corresponding number of locus pairs are extracted according to the sampling count of each distance. After merging, the total number of negative samples is adjusted to about three times of the positive samples. The position bias is eliminated by random rearrangement to form the final negative sample set, which provides support for model training in collaboration with the positive samples.

(4) Loss function and training strategy.

Since the prediction of chromatin loops is essentially a binary classification task (that is, to distinguish between “chromatin loops” and “non chromatin loops”), TM-Loop uses *binary_crossentropy* loss function to optimize the model parameters. Its mathematical definition is as follows:

30 $$\:\begin{array}{c}Binary\:Cross-Entropy\:Loss=-\frac{1}{N}\sum\:_{i=1}^{N}\left[{y}_{i}\cdot\:\mathrm{log}\left({p}_{i}\right)+\left(1-{y}_{i}\right)\cdot\:\mathrm{log}\left(1-{p}_{i}\right)\right]\end{array}$$

Where *N* is the total number of samples, *y*_*i*_ represents the real label of the ith sample, and *p*_*i*_ is the prediction probability of the ith sample output by the model. In order to improve the stability and convergence efficiency of model training, Adam optimizer^36,37^ is used to implement parameter iterative update, and the ReduceLROnPlateau strategy^38^ is introduced to dynamically adjust the learning rate. When the loss of verification set has not decreased significantly for several consecutive rounds, the learning rate is automatically reduced until it reaches the preset minimum value. During the training process, the model continues to retain the parameter combination with the best performance of the verification set for the performance evaluation of subsequent testing links.

## Results

In order to systematically verify the effectiveness and superiority of TM-Loop, a multi-level evaluation system is constructed. The specific scheme is as follows: firstly, the basic prediction performance of TM-Loop is preliminarily verified by using independent test set samples; Taking complete multi chromosome samples as the research object, TM-Loop was compared with existing mainstream chromatin loop prediction methods such as Mustache, Chromosight, Peakachu, DloopCaller to highlight its performance advantages.

In order to further verify the universality and robustness of the method, a comprehensive performance evaluation was carried out on TM-Loop in two different cell lines, GM12878 and K562. The evaluation dimensions include aggregation peak analysis (APA), binding factor enrichment analysis, promoter enhancer interaction correlation analysis, chromatin loop overlap rate analysis, loop distance distribution analysis and other core evaluation indicators. The application value of TM-Loop in accurate detection of chromatin loop was comprehensively demonstrated from multiple dimensions, such as biological rationality, prediction accuracy, and method universality.

### Model checking

TM-Loop carried out the test on chromosomes 15, 16 and 17 of gm12878 cell line by chromosome retention method. In the experiment, the model and parameter combination with the best performance are loaded, and the above test set samples are input into the model to complete the prediction analysis.

This study selected Accuracy, Precision, Recall, F1 score, ROC AUC and Matthews correlation coefficient (MCC) as the evaluation indicators of model performance^39,40^.

**Fig. 2:**
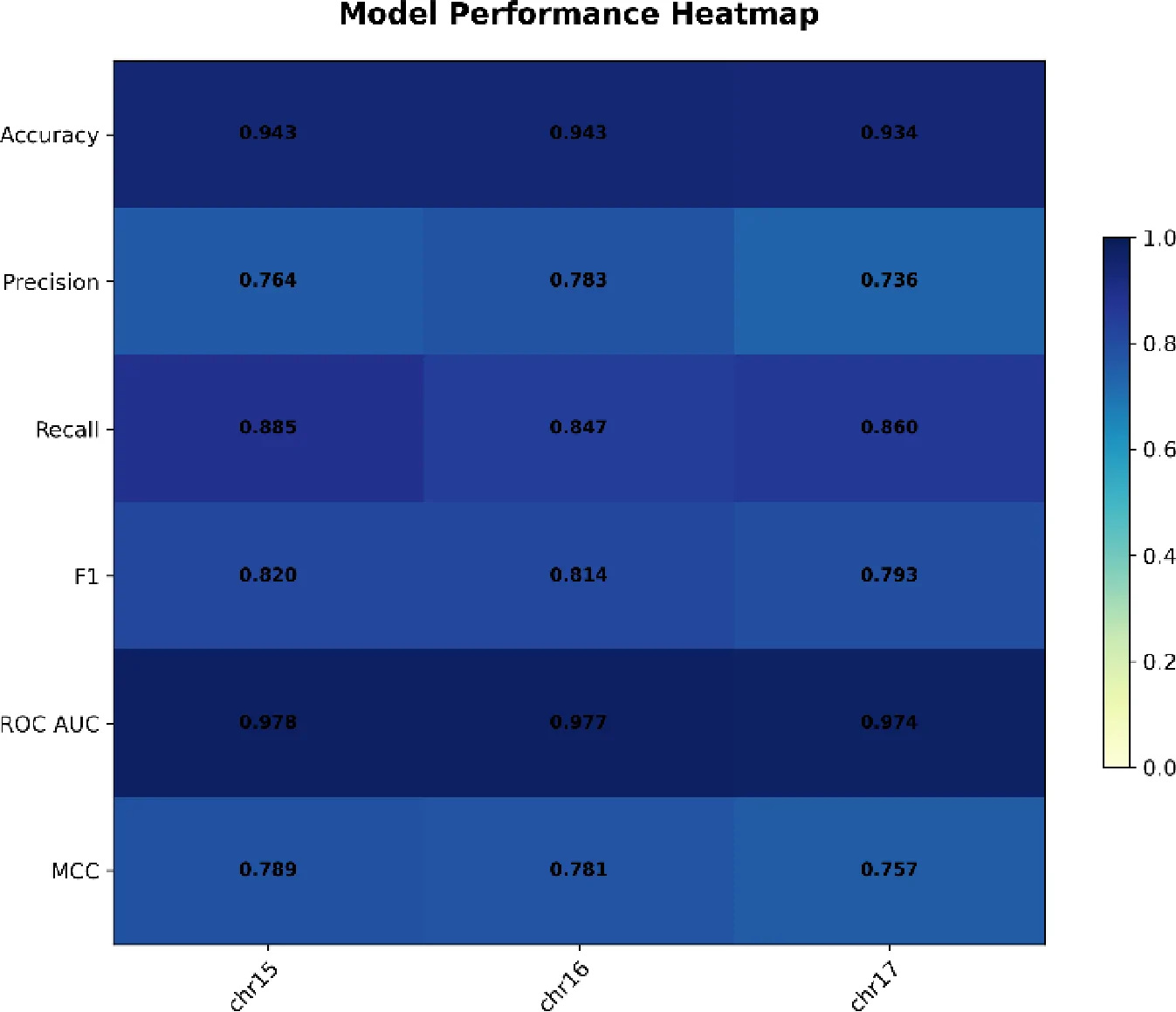
TM-Loop model performance heat map.

From the model performance heat map, TM-Loop showed stable and excellent prediction performance on the three tested chromosomes: the accuracy rate reached 0.943 on chr15 and chr16 chromosomes, and 0.934 on chr17 chromosomes; ROC AUC index is particularly outstanding, with the highest value of 0.978 on chr15, 0.977 on chr16, and 0.974 on chr17, showing the model’s strong ability to distinguish positive and negative samples; The recall rate reached 0.885 on chr15, reflecting the high capture efficiency of the model for positive samples; At the same time, the F1 value is between 0.793 and 0.820, and the MCC is between 0.757 and 0.789, which reflects that the model has achieved a good balance between accuracy and recall Fig. 2.

The results show that TM-Loop shows stable and accurate prediction performance on the independent chromosome test set without training, and verifies the reliability and generalization ability of the model.

### Ablation study of key modules in TM-Loop

To verify the effectiveness and necessity of each core component in TM-Loop, we conducted systematic ablation experiments on the chr15 test set of GM12878 cell line. All ablation groups maintained identical model structure, training parameters and data preprocessing pipelines, and model performance was evaluated by Accuracy, F1-score, AUC, MCC and PR AUC.

To quantify the performance gain brought by different omics data in chromatin loop detection, we designed four comparative groups: only Hi-C handcrafted features, Hi-C + ATAC-seq features, Hi-C + CTCF ChIP-seq features, and full multi-omics integration model (Hi-C + ATAC-seq + CTCF ChIP-seq). The quantitative results are listed in Table 1.

The ablation results demonstrate the synergistic effect of multi-omics data. The model using only Hi-C features achieves baseline performance (F1-score = 0.7983, AUC = 0.9742), providing a reference for performance improvement. Adding ATAC-seq features slightly enhances all metrics, indicating that chromatin accessibility information supplements the contextual features of chromatin interactions. Incorporating CTCF ChIP-seq features leads to a more significant performance boost (F1-score = 0.8193, MCC = 0.7874), confirming that CTCF binding signals play a critical guiding role in chromatin loop identification. The full multi-omics integration model reaches the optimal performance across all indicators, which validates the effectiveness and necessity of the multi-modal fusion strategy proposed in this study.

To verify the improvement of feature utilization efficiency by the feature weighting module, we set two comparative groups on the chr15 test set: without feature weighting (equal weight for all features) and with feature weighting (full model). The performance comparison is shown in Table 2.

The feature weighting mechanism assigns weights according to the contribution of different features to chromatin loop recognition, which suppresses the interference of redundant features and strengthens the expression of key features. Compared with the equal-weight model, the full model with feature weighting improves F1-score from 0.8127 to 0.8198 and MCC from 0.7806 to 0.7889, with slight increments in AUC and PR AUC. These results confirm that the feature weighting mechanism optimizes the utilization efficiency of multi-omics and handcrafted features, and is an essential component for improving model recognition performance.

The Transformer encoder consists of two core components: positional encoding (providing positional information for feature sequences) and multi-head attention (modeling long-range dependencies of chromatin interactions). We designed three ablation groups on the chr15 test set: only remove positional encoding, only remove multi-head attention, and full model (with both components). The performance results are presented in Table 3.

The two core components of the Transformer encoder are indispensable and show a significant synergistic effect. Removing positional encoding causes a sharp drop in model performance (F1-score = 0.4742, PR AUC = 0.5421). Positional encoding provides critical location information for feature sequences; without it, the multi-head attention mechanism cannot perceive the sequence order and relative position of chromatin interaction features, leading to a severe loss of spatial association modeling ability. Removing multi-head attention also results in an obvious performance decline (F1-score = 0.6829, MCC = 0.6309), but the decline is milder than that of removing positional encoding. This indicates that multi-head attention is the core component for capturing long-range interaction dependencies, and its absence significantly weakens the model’s ability to model global context. The full model achieves the best performance by leveraging the synergy of positional encoding and multi-head attention, which fully mines the sequence position information and long-range correlations of chromatin interaction features. These results verify the necessity and effectiveness of the Transformer encoder module and its two core components. Other related model experiments are presented in Supplementary File 1.

**Table 1:** Model performance under different multi-omics integration strategies (chr15 test set).

Experimental group	Accuracy	F1-score	AUC	MCC	PR AUC
Only Hi-C handcrafted features	0.9353	0.7983	0.9742	0.7635	0.8921
Hi-C + ATAC-seq feature fusion	0.9373	0.8037	0.9751	0.7698	0.8975
Hi-C + CTCF ChIP-seq feature fusion	0.9448	0.8193	0.9753	0.7874	0.8983
Full multi-omics integration model	0.9427	0.8198	0.9779	0.7889	0.9073

**Table 2:** Model performance with or without feature weighting mechanism (chr15 test set).

Experimental group	Accuracy	F1-score	AUC	MCC	PR AUC
Without feature weighting (equal weight)	0.9399	0.8127	0.9768	0.7806	0.9053
With feature weighting (full model)	0.9427	0.8198	0.9779	0.7889	0.9073

**Table 3:** Ablation performance of core components in Transformer encoder (chr15 test set).

Experimental group	Accuracy	F1-score	AUC	MCC	PR AUC
Only remove positional encoding	0.8651	0.4742	0.8510	0.4047	0.5421
Only remove multi-head attention	0.8866	0.6829	0.9367	0.6309	0.7637
Full model (positional encoding + multi-head attention)	0.9427	0.8198	0.9779	0.7889	0.9073

### Aggregation peak analysis (APA) results

Aggregation peak analysis (APA) is a classical analytical method for qualitative identification and quantitative evaluation of chromatin interaction signal aggregation peaks. The “aggregation peak” refers to the regions with significantly increased interaction signals in the genome, which are usually highly coincident with the enrichment regions of gene regulatory elements such as enhancers and promoters^41^. APA analysis can systematically reveal the spatial aggregation rule of chromatin, and its core quantitative index APA score is used to characterize the difference between the central signal area and the background signal. The calculation method is: divide the average contact frequency of the central area of the interaction matrix of a specific size by the average contact frequency of the background area in the lower left corner of the matrix.

**Fig. 3:**
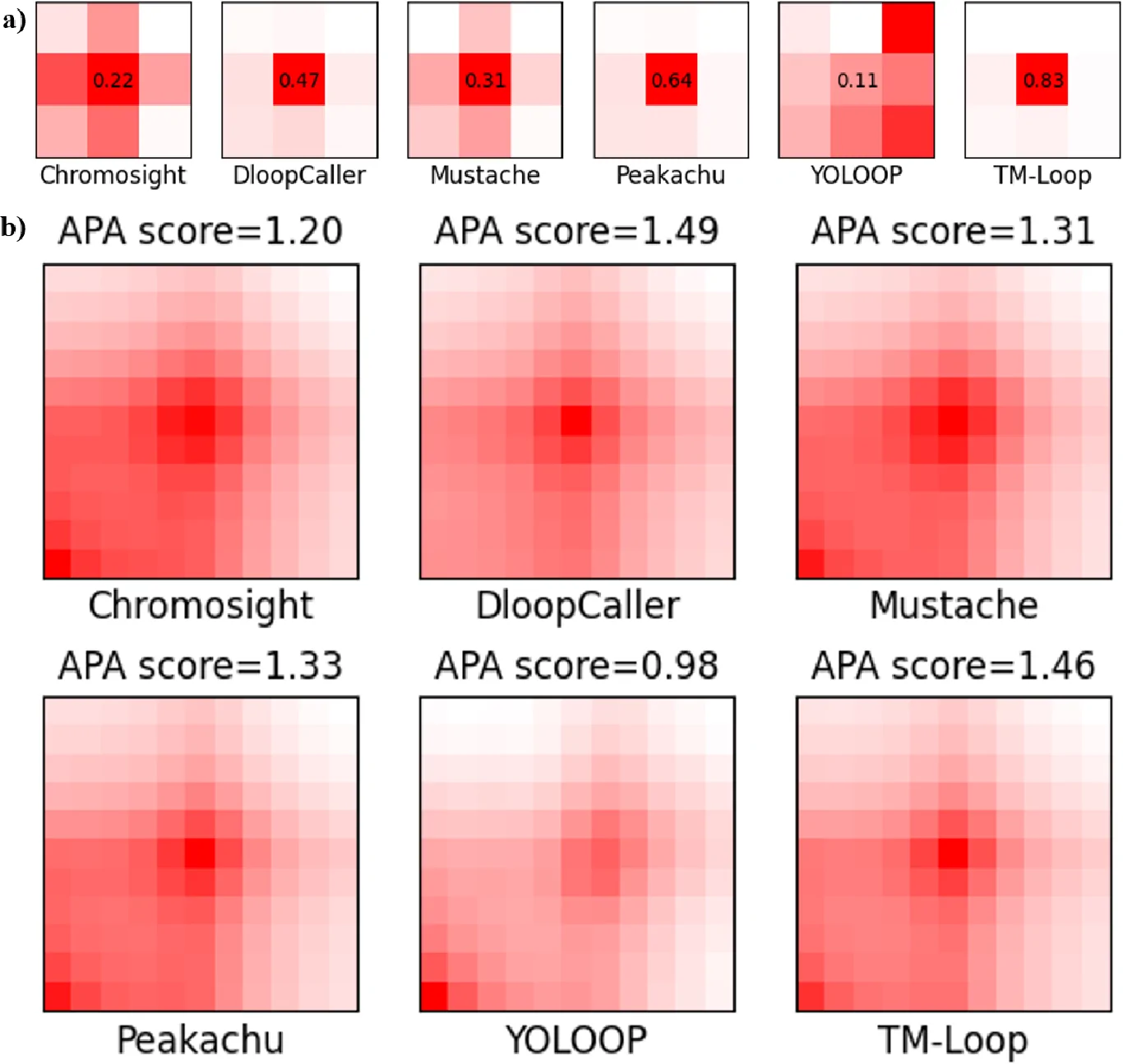
Aggregation peak analysis.

In order to accurately evaluate and predict the local signal enrichment in the central region of the loop, we first analyzed the signal strength of the eight background pixels around the anchor point of the loop, and quantified the significance of local signal enrichment by calculating the intensity ratio of the central pixel to the surrounding pixels. From the local signal ratio diagram below, it can be seen that the signal ratio between the central pixel of the TM-Loop prediction loop and the surrounding background is 0.83, which is significantly higher than Chromosight(0.22), Dloopcaller(0.47), Mustache(0.31), Peakachu(0.64), And YOLOOP (0.11) and other tools, this result shows that the center of the chromatin loop predicted by TM-Loop has a stronger local signal enrichment effect, which is closer to the high signal peak characteristics of the real chromatin loop, the results are shown in Fig. 3(a).

In order to further verify the global spatial aggregation characteristics of different tool prediction loops, we construct an 11 × 11 size interaction matrix for APA analysis, and the calculated APA score can reflect the overall signal difference level between the central region and the background region. From the global APA score results below, the APA score of TM-Loop is 1.46, which is equivalent to the performance of Dloopcaller (1.49), but significantly higher than that of Chromosight (1.20), Mustache (1.31), Peakachu (1.33), YOLOOP (0.98) and other tools, suggesting that the chromatin loop predicted by TM-Loop has a more significant central signal aggregation effect on the global scale, and its spatial aggregation characteristics are more consistent with the biological laws of the real chromatin loop, the results are shown in Fig. 3(b).

Combining the analysis results of local signal ratio and global APA score, TM-Loop is outstanding in predicting the signal significance and spatial aggregation characteristics of the chromatin loop, which further verifies the biological reliability of the prediction results of the tool.

### Enrichment validation analysis of key regulatory factors

As the core transcription factor in the regulation of chromatin three-dimensional conformation, CTCF can form an anchor structure by combining with genome-specific sites, and interact with enhancers and other remote regulatory elements in space^42,43^, so as to narrow the DNA fragments in different regions and mediate the assembly of chromatin loops. H3K27ac, as a histone modification marker of the active regulatory region^44^, is mainly enriched in regulatory elements with transcriptional activity such as enhancers and promoters. Its distribution in the chromatin loop can directly reflect the transcriptional regulatory activity of this region^45,46^. RAD21 and SMC1 are the core subunits of the adhesin complex, responsible for maintaining the high-level folding structure of chromatin, and promoting the formation and stable maintenance of chromatin loop by anchoring and aggregating DNA fragments in different regions^47,48^.

In order to verify the biological reliability of the prediction results, we selected chromosomes 15, 16 and 17 of GM12878 cell line as the test area, and analyzed the correlation between the prediction loops of each tool and the target loops verified by experiments such as CTCF ChIA-PET, SMC1 HiChIP, RAD21 ChIA-PET^49^, H3K27ac HiChIP, etc. We performed cross-resolution integration and redundancy removal on the above multiple sets of experimental data to cover chromatin loops of different functional types, and used the matching algorithm provided by RefHiC^50^ to quantitatively calculate the coincidence degree between the prediction loop and the experimental target loop.

**Fig. 4:**
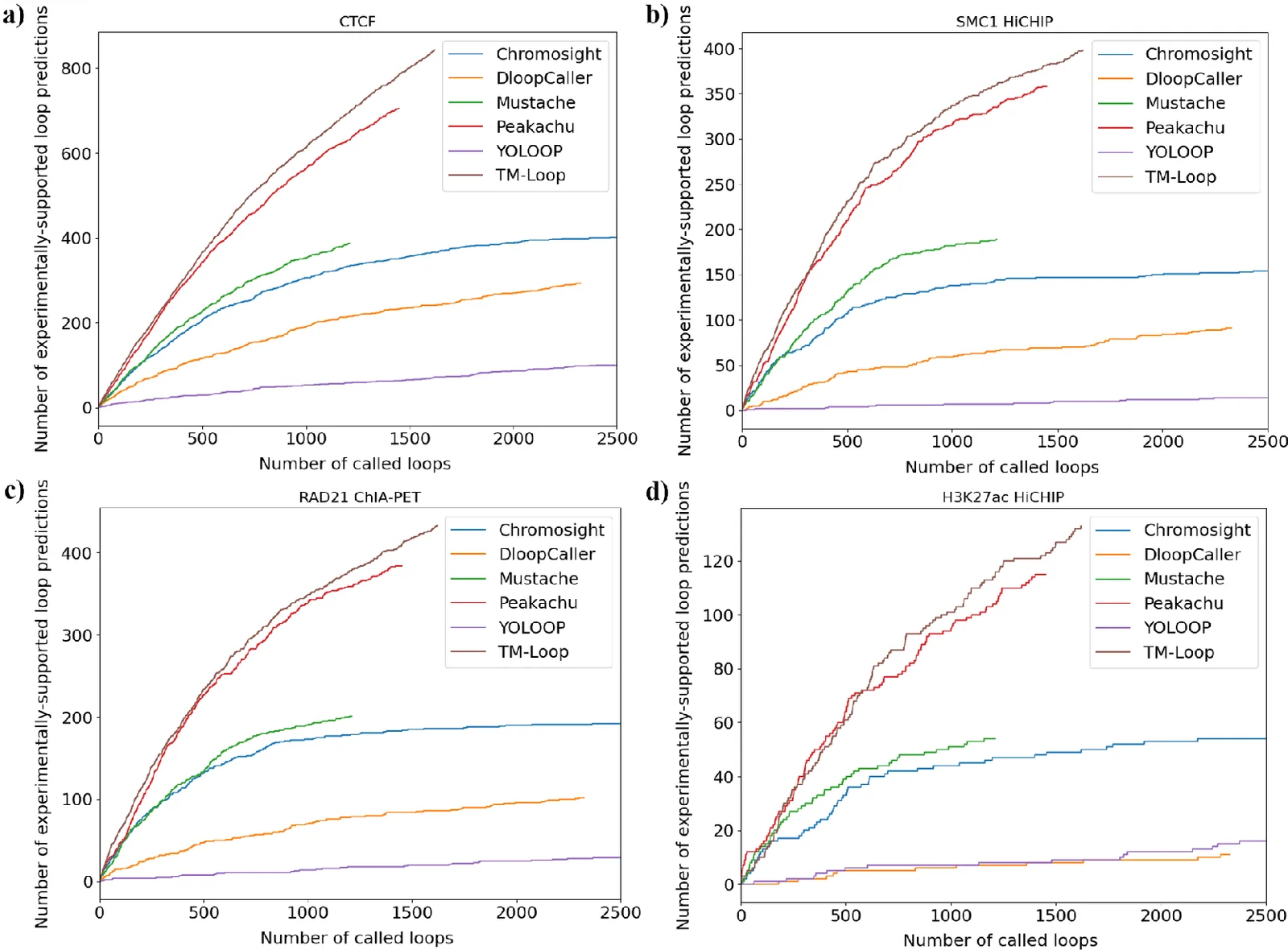
Enrichment factor analysis of structural proteins integrated from CTCF ChIA-PET, H3K27ac HiChIP, SMC1 HiChIP and RAD21 ChIA-PET data.

The results of correlation analysis showed that the experimental support of TM-Loop was the best among the target loop validation of all regulatory factors. In the CTCF ChIA-PET verification (Fig. 4 (a)), with the increase of the number of prediction loops, the number of experimental verification loops corresponding to TM-Loop is always significantly higher than that of Chromosight, Dloopcaller, Mustache, Peakachu and YOLOOP. Even when 2500 loops are predicted, it still remains in the lead, indicating that it has efficient recognition ability for CTCF mediated chromatin loops. In the verification of SMC1 HiChIP (Fig. 4 (b)) and RAD21 ChIA-PET (Fig. 4 (c)), the performance curve of TM-Loop is also in the leading position, and the number of experimental support loops obtained under the same predicted number is the largest, which further confirms its ability to accurately capture the functional loops related to the adhesin complex. For the H3K27ac HiChIP labeled active regulatory element loop (Fig. 4 (d)), the number of experimental support loops of TM-Loop is also significantly higher than that of other tools, reflecting its high sensitivity recognition characteristics for functional chromatin loops such as enhancer promoter.

In conclusion, TM-Loop has the highest experimental support in the target loop enriched with specific regulatory factors, and the number of matching loops between prediction and experimental identification is the largest, indicating that the prediction results of this tool have better biological consistency.

### Analysis of anchor enrichment characteristics of CTCF binding sites

In the association analysis of CTCF binding sites, the chromatin loop is often presented in the form of peak signals on the interaction map drawn by Hi-C technology, and the positions of these peaks correspond to the anchor region of the chromatin loop. The height of the CTCF ChIP-seq signal peak directly reflects the CTCF binding strength of the site. The higher the binding strength of the genomic region, the greater the probability of participating in chromatin loop assembly^51^. Therefore, we evaluated the biological reliability of the prediction results of different tools by quantifying the enrichment ratio of CTCF binding peaks in the chromatin loop anchor and its flanking region predicted by different tools.

**Fig. 5:**
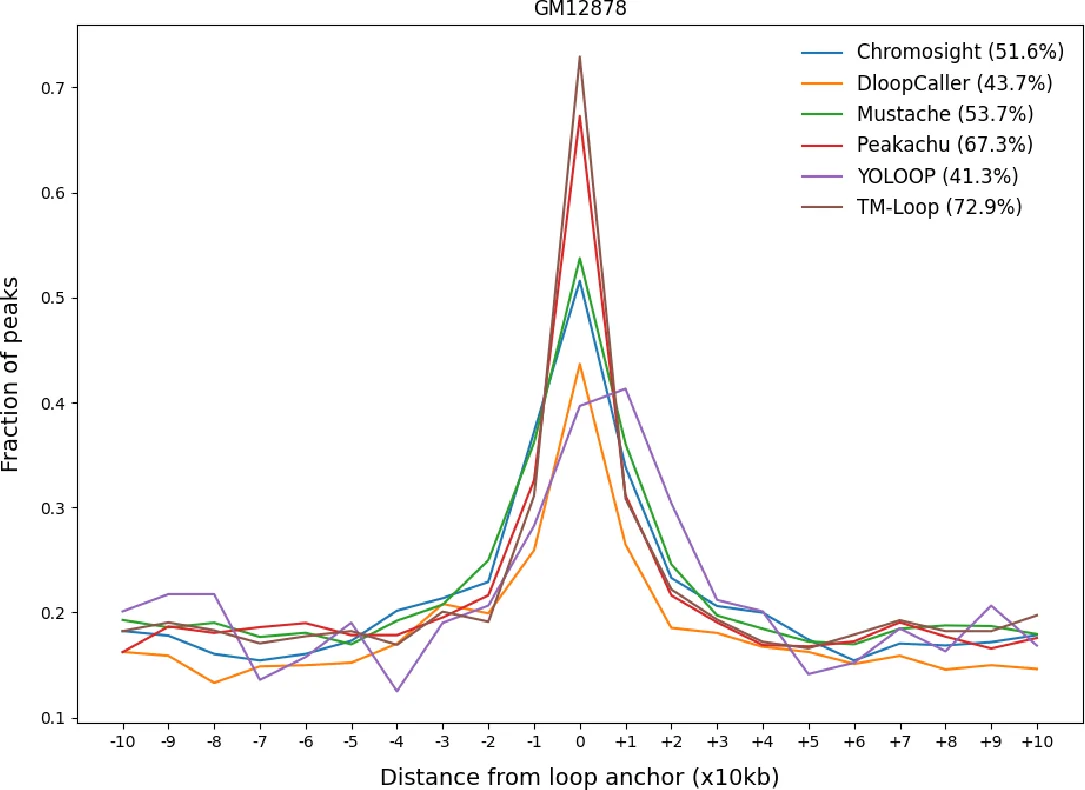
In the gm12878 cell line, the CTCF peak was located near the anchor point of the chromatin loop, which was determined by different tools.

It can be seen from the figure that the chromatin loop anchors predicted by all tools show significant peak enrichment characteristics at CTCF binding sites and their flanking regions, indicating that these tools can recognize a large number of loop anchors related to CTCF binding sites. Among them, the enrichment ratio of CTCF binding peak of TM-Loop was the highest, reaching 72.9%, which was significantly higher than that of Chromosight (51.6%), DloopCaller (43.7%), Mustache (53.7%), Peakachu (67.3%), and YOLOOP (41.3%). Notably, YOLOOP exhibits the lowest CTCF peak enrichment ratio among all compared methods, indicating that the correlation between the loop anchor predicted by Fig. 5.

TM-Loop and CTCF binding site was stronger, which further verified the reliability of the prediction results. Other results of GM12878 cell line are supplemented in Supplementary File 1.

### Cross Cell-Type and Cross-Species Verification in K562, H1ESC and mESC Cell Lines

Although this model was only trained on Hi-C data of the human lymphoblastoid cell line GM12878, we further evaluated its generalization performance across multiple biological contexts, including the human hematopoietic malignant cell line K562, the mouse embryonic stem cell line mESC, and the human embryonic stem cell line H1ESC.

**Fig. 6:**
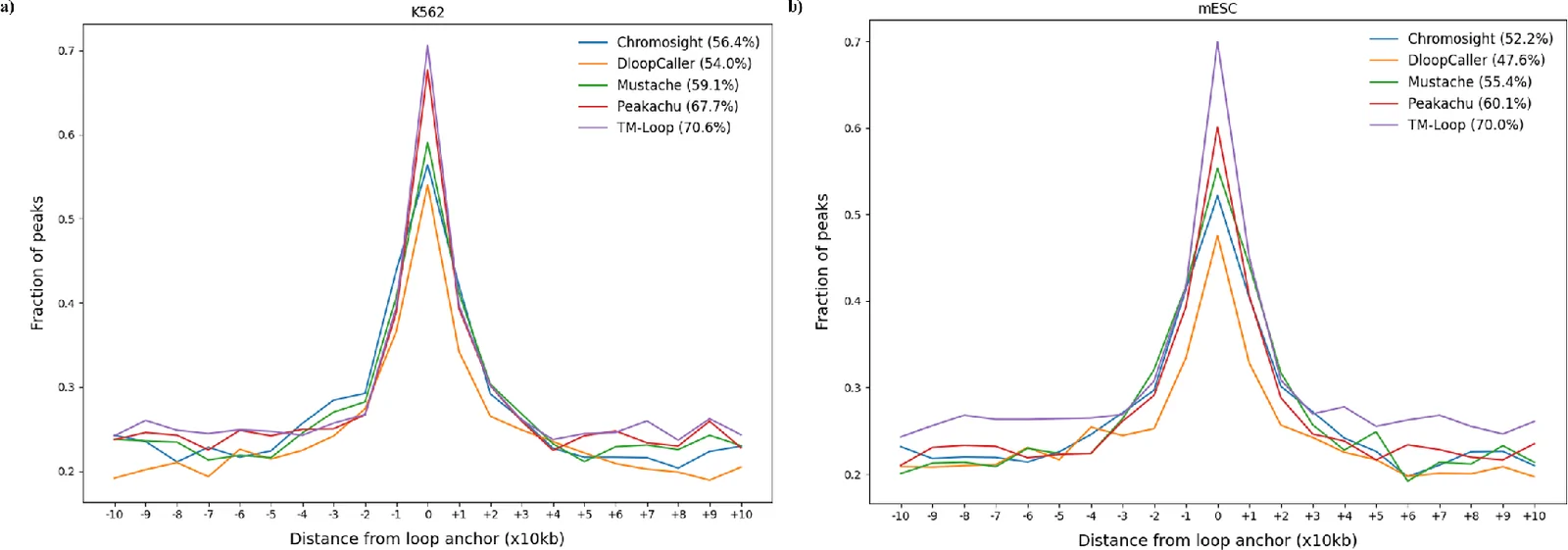
CTCF peaks near chromatin loop anchor points identified by different tools in K562 and mESC cell lines.

For the K562 cell line, a classic model of chronic myeloid leukemia with significant differences in three-dimensional chromatin conformation compared to normal B cells, we selected chromosomes 15–17 as an independent test set to compare the detection efficiency of TM-Loop with existing mainstream algorithms. According to the analysis of CTCF binding site enrichment patterns around loop anchors (Fig. 6 (a)), loop anchors predicted by TM-Loop showed sharp CTCF signal peaks within the ± 100 kb window, with a peak enrichment ratio of 70.6%. This value was significantly higher than that of Chromosight (56.4%), Mustache (59.1%), and Peakachu (67.7%), while DloopCaller showed the weakest enrichment at 54.0%, further verifying TM-Loop’s preferential recognition of structural protein-anchored chromatin loops.

To further assess cross-species generalization ability, we extended our evaluation to the mouse embryonic stem cell line mESC, which has distinct three-dimensional chromatin organization compared to human cell lines. CTCF binding site enrichment analysis of loop anchors predicted by TM-Loop and competing methods in mESC (Fig. 6 (b)) revealed that TM-Loop-identified anchors exhibited the most prominent and sharp CTCF signal peak within the ± 100 kb window, with a peak enrichment ratio of 70.0%. This performance was significantly higher than all compared methods, including Chromosight (52.2%), DloopCaller (47.6%), Mustache (55.4%), and Peakachu (60.1%). Notably, TM-Loop maintained a certain correlation with CTCF binding sites even across species, demonstrating its certain degree of stability in identifying evolutionarily conserved structural chromatin loops.

In conclusion, although trained exclusively on the human lymphoblastoid cell line GM12878, TM-Loop exhibits certain generalization performance across multiple human cell lines and mouse cell models. It maintains detection accuracy comparable to the training set in the heterogeneous K562 cell line, achieves stable zero-shot cross-cell-line performance in the human embryonic stem cell line H1ESC, and retains good performance in identifying CTCF-associated structural loops even in the cross-species mESC model. These results suggest that TM-Loop has certain cross-cell-line and cross-species generalization capabilities, indicating its potential applicability for chromatin loop detection across diverse biological contexts. Additional results for K562, H1ESC, and mESC cell lines are provided in Supplementary File 1.

## Discussion

Although TM-Loop has reliable and robust performance in detecting chromatin loops from Hi-C contact maps, there are still non-negligible limitations in this work that need to be acknowledged and improved in future studies.

On the algorithm level, the multi-dimensional combined feature system adopted in this work ensures stable detection performance for chromatin loops, but the step-by-step window traversal, multi-omics feature extraction and large-scale candidate region inference bring relatively high computational cost. Under the background of continuous improvement of Hi-C sequencing resolution and rapid expansion of sequencing sample size, the time overhead of full-resolution prediction gradually becomes a limiting factor for efficient data analysis. In the future, we will introduce block-based segmentation processing, model lightweight optimization and batch parallel inference strategies to reduce runtime and hardware resource consumption, so as to realize efficient and rapid mining of high-resolution and large-scale chromatin interaction data.

On the data dimension of multi-omics integration, our model only utilizes ATAC-seq open chromatin signals and CTCF binding profiles to construct epigenetic auxiliary features. However, chromatin loop formation is regulated by multiple layers of epigenetic modifications. The lack of key histone modification data including H3K27ac, H3K4me3 limits the comprehensive characterization of regulatory patterns. Expanding the types of input epigenetic data will help enrich model feature dimensions, weaken the bias of single-omics data, and improve the biological comprehensiveness and cell-type specificity of prediction results.

## Conclusion

The chromatin loop prediction method based on a variety of three-dimensional genome modification data provides the core technical support for the analysis of three-dimensional genome spatial organization and gene regulation mechanism. Traditional chromatin loop detection methods often rely on a single Hi-C data type, which is easily affected by data noise, imbalance of positive and negative sample categories, resulting in high false positive rate and insufficient biological correlation. The combination of multi omics data integration and deep learning technology provides a new direction to solve the above problems. In this study, we developed a high-precision, low false-positive chromatin loop detection method TM-loop, which takes the 10 kb resolution Hi-C contact matrix as the core, integrates ATAC-seq、CTCF ChIP-seq and other three-dimensional genome modification data, and realizes the accurate identification of chromatin loops through the four stage core process.

The core process of TM-loop revolves around the efficient use of multiomics data and the optimization of prediction results layer by layer: first, in the stage of multiomics data integration and standardization, the method first integrates the heterogeneous data of Hi-C, ATAC-seq and CTCF ChIP-seq, completes KR normalization and sparse processing for Hi-C data to eliminate technical deviation, carries out windowed extraction and missing value filling for ATAC-seq、CTCF ChIP-seq and other epigenetic data, and constructs a balanced positive and negative sample set to effectively alleviate the interference of class imbalance on model training; Secondly, in the construction phase of multi-dimensional weighted features, for each preprocessed genome window region, multi-dimensional features such as Hi-C derived structural features and epigenetic modification features are extracted, and the organic fusion of multi-source features is realized through the differentiated weighting strategy, which is transformed into a unified 516 dimensional feature vector, giving full play to the role of multi-data complementary verification and effectively eliminating the noise interference of single data; Thirdly, in the generation stage of high confidence candidate chromatin loops, 516 dimensional feature vectors are trained based on the pre training deep learning framework to output the prediction probability of chromatin loops, and the high confidence candidate chromatin loops are further screened through the double threshold screening strategy, which significantly reduces the false positive rate of the prediction results; Fourthly, in the hierarchical multi-scale clustering optimization stage, the adaptive density hierarchical multi-scale clustering algorithm is used to optimize the candidate chromatin loops, eliminate redundant candidate loops, and finally output high-quality, non redundant chromatin loop data sets.

In order to verify the effectiveness and superiority of TM-loop, we carried out multi-dimensional evaluation experiments: the biological rationality of the prediction results was verified by aggregation peak analysis (APA) and binding factor enrichment analysis. The results showed that the binding sites of the chromatin loop recognized by TM-loop and key regulatory factors such as CTCF were highly enriched, which had significant biological significance; Compared with mainstream chromatin loop detection tools such as Mustache, Chromosight, Peakachu and DLoopCaller, TM-loop showed obvious advantages in false positive rate control and prediction accuracy; At the same time, it was verified in different cell lines that the chromatin loop predicted by this method has good consistency and robustness across cell lines, reflecting excellent versatility.

## Supplementary Information

Below is the link to the electronic supplementary material.

Supplementary Material 1

## Data Availability

All data used in this paper are shown in the Supplementary File 1.
