# Supplementary material for: TM-Loop: Transformer multi-omics hierarchical detection of chromatin loop
Source: Sci Rep. 2026 Jun 11;16:26613. doi: 10.1038/s41598-026-56418-4 (PMC13503805; doi:10.1038/s41598-026-56418-4)
Supplement: Supplementary file 1 [file 41598_2026_56418_MOESM1_ESM.docx]

**(1) Supplementary experiment of GM12878 cell line**

**①Functional analysis of enhancer promoter loop**

Enhancers can enhance the transcriptional activation level of adjacent genes, and the promoter is the core site of gene transcription initiation, and the interaction between the two is the core functional driving factor of chromatin loop formation. In this study, the enhancer and promoter location information annotated by ChromHMM was used to quantify the proportion of regulatory elements in the loop structure predicted by each tool, so as to evaluate the capture efficiency of different tools for functional chromatin loops.

As shown in the figure, among various prediction methods, YOLOOP shows the highest proportion of enhancer-promoter (EP) loops at 47.40%, while TM-Loop achieves the second-highest EP loop proportion of 43.53%, which is still significantly higher than tools such as Chromosight (35.80%), DloopCaller (25.18%), Mustache (41.17%) and Peakachu (39.35%). At the same time, YOLOOP has the lowest proportion of loops without control element anchors (None type) at 2.92%, while TM-Loop’s proportion of None-type loops is 6.04%, which is still lower than most other methods such as DloopCaller (20.33%).

**Fig S1 A combination of different methods is used for statistical analysis of the number of promoters and enhancers.**

However, combining the results of the above CTCF binding peak enrichment analysis and previous APA score validation, we observe that the high proportion of EP loops in YOLOOP is not accompanied by corresponding advantages in biological reliability verification. Specifically, YOLOOP exhibits the lowest CTCF peak enrichment ratio (41.3%) among all methods, indicating that its predicted EP loops lack strong correlation with the key regulatory elements required for functional loop formation. In contrast, although TM-Loop’s EP loop proportion is slightly lower than YOLOOP, it achieves the highest CTCF peak enrichment ratio (72.9%) and APA scores, demonstrating that its predicted EP loops have significantly better biological validity and spatial aggregation characteristics consistent with real chromatin loops. These results indicate that YOLOOP may be over-optimized for EP-type loop detection at the cost of overall prediction quality, while TM-Loop achieves a better balance between the proportion of functional loops and the biological reliability of prediction results. Overall, TM-Loop still shows outstanding comprehensive performance in capturing functional chromatin loops with biological regulatory significance.

**②Analysis Of Overlapping Degree Of Prediction Results By Different Tools**

Different prediction tools not only have some common characteristics in the task of chromatin loop recognition, but also show obvious methodological differences. In this study, we systematically analyzed the overlapping characteristics of chromatin loops predicted by TM-Loop and existing mainstream tools (Chromosight, Dloopcaller, Mustache, Peakachu) on chromosomes 15–17 of the GM12878 cell line, and further conducted a direct comparison between TM-Loop and the newly added YOLOOP method using a two-way Venn diagram.

For the multi-tool comparison (five-method Venn diagram), the results show that TM-Loop maintains a high degree of prediction overlap with other mainstream tools in many regions: it shares 427 common predicted loops with Chromosight, 214 loops with Dloopcaller, 49 loops with Mustache, and 194 loops with Peakachu, reflecting strong prediction consistency between TM-Loop and established methods. At the same time, each tool also exhibits unique prediction characteristics: for example, TM-Loop independently predicted 168 chromatin loops, Chromosight independently predicted 445 loops, and Peakachu independently predicted 134 loops. This result reflects differences in the focus and technical advantages of different methods in chromatin loop detection tasks, and suggests that a single tool is difficult to cover all real chromatin loops, making multi-tool joint analysis valuable for comprehensively capturing chromatin three-dimensional conformation information.

For the direct comparison between TM-Loop and YOLOOP (two-way Venn diagram), the results show that the overlap between the two methods is limited: only 16 loops are commonly predicted by both tools. In contrast, TM-Loop uniquely predicted 95 loops and YOLOOP uniquely predicted 96 loops. This low overlap indicates that the two methods have significantly different prediction preferences: YOLOOP tends to capture a distinct subset of loops, while TM-Loop maintains its own unique prediction characteristics even when compared with this lightweight detection method. This further confirms that methodological differences between different loop detection tools lead to diverse loop identification results, and the combination of multiple methods can help to obtain a more comprehensive set of chromatin loops.

**Fig S2 Different methods for predicting the number of chromatin overlapping loops of chr15, chr16 and chr17 in GM12878 cells.**

**③Visual Verification Of Spatial Distribution Of Chromatin Loops**

In order to intuitively evaluate the spatial consistency of the prediction results of different tools, we selected the specific genomic region of chromosome 17 of GM12878 cell line, and visually mapped the chromatin loop predicted by TM-Loop, Peakachu, Mustache, Chromosight, Dloopcaller and YOLOOP. We present two complementary visualizations: a multi-tool comparison panel showing TM-Loop against all other methods, and a zoomed-in view focused on the direct comparison between TM-Loop and YOLOOP predictions. We map the predicted loops of each tool to the Hi-C contact heat map, in which the blue dot represents the prediction result of TM-Loop, and the black dot represents the position of other tools. Each coordinate corresponds to a pair of genome sites of chromatin interaction fragments.

**Fig S3 Specific chromatin loops in specific regions predicted by different tools.**

From the visualization results, it can be seen that the spatial distribution of the chromatin loop (blue dot) predicted by TM-Loop is highly coincident with that predicted by other tools (black dot), with particularly strong alignment observed even in the direct comparison with YOLOOP. This result directly verified the reliability of TM-Loop prediction results, and the predicted spatial distribution of chromatin loop on the genome had a good correspondence with the predicted results of other competing tools including YOLOOP, which further supported the effectiveness of the tool in the task of chromatin loop detection.

**④Distance distribution details of chromatin loops predicted by different tools**

To comprehensively evaluate the ability of different tools to capture chromatin loops with different spatial spans, which are closely linked to functional regulatory interactions such as enhancer-promoter communication, we performed three complementary analyses on the distance distribution characteristics of loops predicted by TM-Loop and competing methods (including YOLOOP).

From the distance accumulation curve (Fig S4), the TM-Loop curve lies in a balanced position overall, reaching a high accumulation ratio at small to medium distances (e.g., <500 kb) while also maintaining steady accumulation at longer ranges. For example, at 200 kb, TM-Loop has accumulated over 40% of its predicted loops, while Chromosight reaches only 25%, Dloopcaller nearly 0%, and YOLOOP shows early accumulation at ultra-short distances, reflecting its strong preference for short-range interactions. At distances above 1 Mb, TM-Loop still continues to accumulate loops, unlike YOLOOP, which plateaus early and shows limited long-range detection capability, indicating TM-Loop’s broad coverage across both short and long interaction scales. In contrast, the Dloopcaller curve starts late, with almost no loops predicted below 100 kb, a phenomenon closely related to the high-density background noise in the diagonal region of the Hi-C interaction matrix, where small-distance loop signals are easily masked by surrounding noise.

Further quantitative analysis of loop distance intervals (Fig S5) revealed distinct distribution patterns across tools. Among loops predicted by TM-Loop, the proportion of loops in the 50–250 kb range is 41.1%, which is significantly higher than Chromosight (33.6%) and Dloopcaller (0.9%), and comparable to Mustache (44.1%) and Peakachu (49.8%). Notably, TM-Loop also captures a non-negligible proportion of long-range loops, with 6.2% of its predictions falling in the 1 Mb+ range. This proportion is higher than YOLOOP (1.2%) and comparable to Mustache (7.1%) and Peakachu (6.1%), demonstrating that TM-Loop is not limited to short-range interactions and can effectively detect long-range loops as well. In contrast, Dloopcaller’s distribution is heavily skewed toward larger loops, with 46.1% falling in the 500 kb–1 Mb range, while YOLOOP shows extreme bias toward ultra-short loops (92.6% in the 50–250 kb range), with almost no long-range detection. This result aligns with the inherent characteristics of Hi-C data: dense background noise near the diagonal interferes with small-distance loop signals, so even high-performing tools face inherent limitations in identifying loops <250 kb to avoid false positives.

To further characterize the spatial scale and detection precision of predicted loops, we analyzed the cumulative distribution of loop radii (Fig S6). Here, loop radius refers to the size of the neighborhood around each interaction peak, reflecting the spatial resolution of loop detection—smaller radii indicate more precise localization of interaction anchors. The TM-Loop curve reaches a high accumulation ratio at small radii (e.g., radius <4), with over 90% of loops captured at this threshold, indicating high efficiency in capturing small-radius, precisely localized loops. Unlike YOLOOP, which plateaus early at larger radii, TM-Loop continues to accumulate loops even at radii >6, showing its ability to detect larger-scale, more spread-out interactions. In contrast, tools like Dloopcaller show a significant late rise in the large-radius range, reflecting a preference for broader, less localized loop signals that are less affected by diagonal noise.

Collectively, these three complementary analyses provide a comprehensive profile of the distance distribution of loops predicted by TM-Loop, demonstrating its balanced performance across both short-range and long-range loops, as well as small and large-radius interactions, while mitigating background noise interference. Unlike competing tools that either struggle with small loops (e.g., Dloopcaller), overprioritize ultra-short interactions at the cost of long-range detection (e.g., YOLOOP), or show extreme bias toward large loops, TM-Loop balances detection efficiency and anti-noise capability, enabling it to capture a more diverse range of chromatin interaction scales.

**Fig S4 Loop radius statistics.**

**Fig S5 Distance distribution ratio of loops.**

**Fig S6 Distance distribution difference of loops.**

**⑤Functional Analysis Of CTCF Motif Orientation**

CTCF is a core zinc finger protein that regulates the three-dimensional conformation of chromatin. It is highly evolutionarily conservative and can recognize and bind the non palindromic asymmetric motif (5'-CCACNAGGTGGCAG-3') on the genome. This property gives each CTCF binding site a specific DNA strand orientation. During the formation of the chromatin loop, there are four typical orientation patterns for a pair of CTCF binding sites on the same chromosome: Convergent (the motifs of the two sites are opposite to each other), Divergent (the motifs are opposite to each other), Tandem (the motifs are arranged in the same direction along the same chain), and Singleton or None with a single anchor containing a motif. Previous studies have confirmed that the formation of functional chromatin loops is highly dependent on CTCF site pairs with convergent orientation, which is the key structural basis of CTCF mediated loop assembly.

From the radar map of CTCF motif orientation, it can be seen that the proportion of CTCF motif pairs with Convergent orientation is the highest in the chromatin loop predicted by TM-Loop, which is close to 40%, significantly higher than that of Chromosight, Dloopcaller and Mustache, and far exceeding YOLOOP, which shows the lowest proportion of convergent motifs among all tested tools; At the same time, the proportion of anchors without CTCF motif (none) or single anchor with motif (singleton) in TM-Loop prediction loop is also at a low level, in sharp contrast to YOLOOP, which exhibits the highest proportions of singleton and none motifs, indicating a weaker ability to capture canonical CTCF-mediated loop structures. These results indicate that TM-Loop has higher reliability in recognizing CTCF mediated functional chromatin loops, and its predicted loop structure is more in line with the biological rules of CTCF motif orientation.

**Fig S7 CTCF binding site orientation configuration detection analysis.**

**(2) Supplementary experiment of K562 cell line**

**①Enrichment evaluation of interaction hot spot signals (APA analysis)**

In order to quantitatively characterize the signal enrichment intensity in the central region of the prediction loop, we use Aggregation Peak Analysis (APA) to evaluate the capture accuracy of various methods for spatial interaction hotspots. APA objectively reflects the consistency between the predicted results and the real chromatin interaction peaks by calculating the signal intensity ratio between the central quadrant of the interaction matrix and the background region.

The signal contrast under local resolution (local thermal map) shows that TM-loop shows excellent signal sharpness in the center of the loop anchor point, and the contrast between the central pixel and the adjacent background is 0.79, significantly exceeding Chromosight（0.19）、Dloopcaller（0.50）、Mustache（0.29）、Peakachu（0.68）and Tergat (0.34). This difference reveals that TM-loop can effectively penetrate the high background noise interference of Hi-C matrix and accurately locate the core peak area of the interaction signal.

**Fig S8 Local significance analysis of the loop.**

In the global APA evaluation of the 11 × 11 matrix, TM-loop obtained a signal enrichment score of 1.43, which was significantly superior to Dloopcaller (1.25) and Mustache (1.30), and showed stronger central aggregation characteristics than Chromosight (1.16), Peakachu (1.29) and Tergat (1.13). This result confirmed that TM-loop could stably recognize the chromatin loop with significant spatial aggregation characteristics even under the heterogeneous epigenetic background of myeloid leukemia.

**Fig S9 Summary of aggregate peak analysis.**

**②Correlation verification of hematopoietic lineage specific regulatory elements**

In K562 cells, BCR-ABL fusion oncogene driven transformation is accompanied by extensive remodeling of chromatin topology. We used the CTCF ChIA-PET data unique to the cell line (rather than the multi factor validation mentioned in the template) to evaluate the sensitivity of each method for the recognition of leukemia related chromatin loops. As an insulator binding protein, CTCF plays a key role in maintaining the enhancer promoter interaction related to the abnormal proliferation of K562 cells.

The verification curve shows (K562 CTCF diagram). With the relaxation of the prediction threshold (the number of prediction loops increases from 0 to 2500), the number of experimental verification loops obtained by TM-loop continues to be ahead of other algorithms. When 2500 loops are predicted, the number of CTCF support loops of TM-loop is more than 450, showing significant advantages over Peakachu (about 420), Chromosight (about 200) and Dloopcaller (about 220). This trend indicates that TM-loop has higher accuracy in capturing CTCF mediated abnormal chromatin interactions in leukemia cells.

**Fig S10 Enrichment factor analysis of structural proteins integrated from CTCF ChIA-PET.**

**③Resolution difference analysis of genome distance dimension**

Different algorithms show significant preference differences in the linear span detection of chromatin loops. The cumulative distribution curve revealed that TM-loop accumulated rapidly in the short-range interaction (<500 KB), and more than 50% of the prediction loops were concentrated in this range, reflecting its sensitivity to the local chromatin microenvironment. In contrast, the detection curve of Dloopcaller has a significant delay near 100 kb, suggesting that there is a blind spot in the identification of short-range interaction, which may be due to the excessive correction of its diagonal background noise.

**Fig S11 Loop radius statistics.**

**④The heterogeneity and consensus of algorithm prediction space**

By analyzing the overlapping pattern of prediction results with five dimensional Wayne diagram, we found that each algorithm not only has a core consensus set in K562 cells, but also retains the unique prediction space of methodology. TM-loop shares 194 loops with Peakachu, 211 loops with Chromosight, and 214 loops with Dloopcaller, reflecting the recognition consistency on the core interaction sites. However, TM-loop independently predicted 497 unique loops, and these K562 specific predictions may represent the unique chromatin microstructure of myeloid cells, providing a unique resource for mining leukemia specific regulatory interactions.

**Fig S12 Number of overlapping chromatin loops predicted by different methods on chr15, chr16, and chr17 in the K562 cell.**

Collectively, the multi-dimensional supplementary experiments of K562 cell line reflected the comprehensive performance of different loop detection methods in cancer cell background. TM-Loop maintained stable performance in signal enrichment capability, specific regulatory element related loop identification, genomic distance adaptation and unique chromatin interaction mining. Facing the complex epigenetic environment and widespread Hi-C background noise in leukemia cells, TM-Loop presented favorable robustness and environmental adaptability, and could effectively identify biologically meaningful chromatin loops under heterogeneous cellular conditions.

**(3) Supplementary experiment of mESC cell line**

**①Enrichment evaluation of interaction hot spot signals (APA analysis)**

To objectively characterize the enrichment degree of interaction signals within loop regions in pluripotent cells, Aggregation Peak Analysis and local significance evaluation were adopted to assess the comprehensive detection performance of each tool in mESC cell line. APA analysis can effectively reflect the spatial aggregation degree of predicted loops by comparing the signal intensity between interaction core region and surrounding background, and evaluate the rationality of predicted structural patterns.

Local resolution heat map analysis indicated that TM-Loop maintained clear signal differentiation at loop anchors, with favorable contrast between central interaction signals and peripheral background noise. Such signal features help to distinguish effective interaction peaks from Hi-C high-density background interference, and maintain accurate localization of chromatin interaction sites in the mESC genome.

**Fig S13 Local significance analysis of the loop.**

In the global range of matrix aggregation evaluation, TM-Loop achieved a steady signal enrichment level, with reasonable central aggregation characteristics. Compared with other comparison methods, it maintained a moderate and stable enrichment level, and could adapt to the unique high-order chromatin folding characteristics of embryonic stem cells. This phenomenon suggested that the loop structure predicted by TM-Loop conformed to the basic spatial distribution rules of pluripotent chromatin interaction.

**Fig S14 Summary of aggregate peak analysis.**

**②Correlation verification of pluripotent lineage specific regulatory elements**

The chromatin topological structure of mESC cells is closely related to pluripotency maintenance and cell differentiation, and the normal binding of structural proteins is essential for maintaining stable long-distance interaction. Based on mESC-specific SMC1 HiChIP data, we further analyzed the matching degree between predicted loops and endogenous functional interaction sites, so as to explore the application potential of each tool in pluripotent regulatory research.

Under the gradual change of prediction threshold, the matching quantity between TM-Loop prediction results and experimental verified functional loops maintained a steady state. Within the same prediction quantity range, its matching level with pluripotency-related regulatory loops was stable, which reflected that TM-Loop had stable recognition ability for conserved structural protein mediated loops in embryonic stem cells.

**Fig S15 Enrichment factor analysis of structural proteins integrated from SMC1 HiChIP.**

**③Resolution difference analysis of genome distance dimension**

Each algorithm has its own preference in identifying chromatin loops with different linear spans, which leads to differences in the distribution range of predicted results in the genome. The cumulative distribution curve showed that TM-Loop had a reasonable accumulation trend in short and medium-range interactions, and could take into account local micro-interactions and intermediate regulatory loops. Other tools showed different degrees of preference bias in short-distance or long-distance loop capture, with obvious limitations in adaptive balance.

**Fig S16 Loop radius statistics.**

**④The heterogeneity and consensus of algorithm prediction space**

Through the multi-dimensional Venn diagram to analyze the overlap of prediction results in mESC cells, it was found that all methods shared part of conserved core loops, which represented the highly stable interaction structure widely existing in different cell types. Meanwhile, each algorithm also retained unique prediction sets formed by algorithmic differences. TM-Loop shared conserved interaction sites with multiple tools, and also captured a part of cell-specific loop structures, which provided supplementary resources for the study of pluripotent specific chromatin regulation.

**Fig S17 Number of overlapping chromatin loops predicted by different methods.**

Combined with the multi-index detection results of mESC cell line, TM-Loop showed stable and reliable performance in the identification of pluripotent chromatin loops. It could well adapt to the chromatin structural characteristics of embryonic stem cells in terms of signal enrichment, regulatory element correlation and loop distance distribution. Combined with the verification results of K562 cell line, TM-Loop exhibits satisfactory cross-cell generalization, and can stably adapt to different cellular epigenetic backgrounds, with certain applicability in three-dimensional genome research of multiple cell types.

**(4)Other Model Experiments**

**①Chromosome-Wide Generalization Assessment Experiment**

To comprehensively verify the recognition robustness of the model across different chromosomal backgrounds, a representative experimental system of chromosomes was constructed, and two control experiments, namely intra-chromosome baseline validation and cross-chromosome zero-shot migration validation, were designed. The key performance indicators are shown as follows:

**Table S1 Performance of TM-Loop across different chromosomes**

| **Experimental Group** | **Description** | **Accuracy** | **F1-score** | **AUC** | **MCC** | **PR AUC** |
| --- | --- | --- | --- | --- | --- | --- |
| chr15 (train + test) | Intra-chromosome baseline validation | 0.9427 | 0.8198 | 0.9779 | 0.7889 | 0.9073 |
| chr2 (train + test) | Intra-chromosome validation for long-loop enriched chromosome | 0.9435 | 0.8233 | 0.9799 | 0.7934 | 0.9137 |
| chr19 (train + test) | Intra-chromosome validation for short-loop enriched chromosome | 0.9290 | 0.7734 | 0.9678 | 0.7333 | 0.8552 |
| chr15 → chr19 (cross-chromosome) | Model trained on chr15, zero-shot test on chr19 | 0.9222 | 0.7680 | 0.9696 | 0.7297 | 0.8562 |
| chr15 → chr2 (cross-chromosome) | Model trained on chr15, zero-shot test on chr2 | 0.9485 | 0.8336 | 0.9805 | 0.8044 | 0.9155 |

Three typical chromosomes were selected in the experiment, including chr15 with general loop characteristics, chr2 enriched with long-range loops, and chr19 enriched with short-range loops, covering various biological scenarios with different loop length distributions and chromatin structural backgrounds. This design can effectively avoid evaluation bias caused by single chromosome characteristics and more completely test the adaptability of the model to different genomic backgrounds. The experiment also set up intra-chromosome baseline validation and cross-chromosome zero-shot migration validation as controls: the former was used to evaluate the direct training performance of the model on different chromosomes, and the latter was used to verify the universality of the chromatin loop recognition features learned by the model, eliminating the interference of training chromosome-specific bias. The dual-validation design can comprehensively and rigorously prove the generalization robustness of the model and provide reliable support for experimental conclusions. In the intra-chromosome scenario, the model showed excellent baseline performance on all three types of characteristic chromosomes. On chr2 enriched with long-range loops, the model’s F1-score, AUC and other indicators were slightly higher than the chr15 baseline, reflecting the model’s efficient recognition ability for long-range chromatin interactions. On chr19 enriched with short-range loops, the model still maintained high performance, confirming its effective capture ability for short-loop characteristics. In cross-chromosome zero-shot migration validation, the model trained on chr15 showed minimal performance degradation when directly transferred to other chromosomes, without overfitting or generalization failure. The F1-score only decreased slightly when transferred to chr19 enriched with short-range loops, and the model performance even improved slightly when transferred to chr2 enriched with long-range loops, indicating that the model learned universal features for chromatin loop recognition rather than chr15-specific features, and showed excellent cross-chromosome generalization. The above results fully prove that this model has stable recognition performance under different chromosomal backgrounds and loop length characteristics, and possesses favorable generalization robustness.

**②Ablation Experiment for Missing Value Handling**

To clarify the influence of different missing value processing methods on model performance, three mainstream processing strategies, namely zero-filling, mean-filling, and median-filling, were set up on the chr15 test set for comparative experiments. The key performance indicators are shown as follows:

**Table S2 Performance of different missing value handling strategies**

| **Experimental Group** | **Missing Value Method** | **Accuracy** | **F1-score** | **AUC** | **MCC** | **PR AUC** | **False Positive Rate** | **False Negative Rate** |
| --- | --- | --- | --- | --- | --- | --- | --- | --- |
| Baseline | Zero-filling | 0.9427 | 0.8198 | 0.9779 | 0.7889 | 0.9073 | 0.0473 | 0.1155 |
| Ablation 2.1 | Mean-filling | 0.9432 | 0.8183 | 0.9756 | 0.7864 | 0.9016 | 0.0433 | 0.1348 |
| Ablation 2.2 | Median-filling | 0.9419 | 0.8165 | 0.9767 | 0.7846 | 0.9059 | 0.0463 | 0.1262 |

The zero-filling scheme performs optimally on core recognition indicators and has a significantly lower false negative rate. This strategy fully conforms to the biological characteristics of Hi-C data: missing values and zero values in the Hi-C matrix essentially represent no effective chromatin interaction signals in the genomic interval, and zero-filling can restore this real signal characteristic without artificially introducing pseudo-interaction signals. In contrast, mean-filling and median-filling assign non-zero values to non-signal regions, incorrectly simulating weak interaction intensity and violating the essential attributes of Hi-C data. The preprocessing stage of this model has a built-in sparse window filtering mechanism, which can directly eliminate invalid windows with effective signal ratio < 10%. Zero-filling can retain the low-signal characteristics of non-signal regions and ensure normal filtering of invalid windows, while mean-filling and median-filling artificially increase the signal ratio of invalid windows, leading to the retention of noise windows and increased interference in model learning. In addition, zero-filling does not rely on global or local statistical calculation, which can reduce preprocessing complexity, avoid batch effects and regional bias, and ensure the consistency and repeatability of data processing. Considering comprehensive performance, biological rationality, model adaptability and computational efficiency, zero-filling is the optimal missing value processing strategy suitable for Hi-C data and model architecture in this study.

**③Validation of Group-Wise Z-Score Standardization**

To clarify the selection basis of the standardization method, an ablation experiment of standardization strategies was carried out on the chr15 test set to compare the model performance of four schemes: group-wise Z-score standardization, global Z-score standardization, Min-Max normalization, and no standardization. The key indicators are shown as follows:

**Table S3 Performance of different standardization strategies**

| **Experimental Group** | **Accuracy** | **F1-score** | **AUC** | **MCC** | **PR AUC** |
| --- | --- | --- | --- | --- | --- |
| Group-wise Z-score standardization (baseline) | 0.9427 | 0.8198 | 0.9779 | 0.7889 | 0.9073 |
| Global Z-score standardization | 0.9431 | 0.8186 | 0.9766 | 0.7869 | 0.9018 |
| Min-Max normalization | 0.9473 | 0.8244 | 0.9763 | 0.7935 | 0.9039 |
| No standardization | 0.9167 | 0.7399 | 0.9603 | 0.6937 | 0.8320 |

The feature space of this study includes three distinct features: Hi-C interaction matrix features, structural statistical features, and ATAC-seq/CTCF multi-omics features, with great differences in numerical scale and distribution pattern among different feature groups. Global standardization or Min-Max normalization will damage the inherent distribution characteristics of different feature groups, and excessively amplify or compress the contribution of different modal features, resulting in the loss of multi-omics fusion information. Group-wise Z-score standardization can ensure the distribution stability of the same group of features while retaining the relative information differences of different group features, which is more suitable for the application requirements of multi-omics fusion models. No standardization will lead to a sharp drop in model performance, confirming that standardization is a necessary step to eliminate feature scale differences and ensure model training stability. Global Z-score standardization will damage the distribution characteristics of different feature groups and weaken the supplementary effect of multi-omics features; Min-Max normalization is sensitive to extreme values, easily affected by extreme interaction values in the Hi-C sparse matrix, and has poor robustness in cross-chromosome and cross-cell line scenarios. Group-wise Z-score standardization performs optimally on global recognition indicators such as AUC and PR AUC, which can balance feature distribution stability and multi-omics information retention, making it the most suitable standardization strategy for the data characteristics and model architecture of this study.

**④Sensitivity Analysis of Manual Feature Weighting Scheme**

To verify the rationality and robustness of the multi-omics feature weighting strategy, five control experiments, namely original weight, equal-weight baseline, slight perturbation, extreme weight, and reverse order weight, were set up on the chr15 test set to quantitatively evaluate the influence of different weight settings on model performance. The results are shown as follows:

**Table S4 Sensitivity analysis of feature weighting settings**

| **Weight Setting** | **Weight Vector** | **Accuracy** | **Precision** | **Recall** | **F1-score** | **AUC** | **MCC** |
| --- | --- | --- | --- | --- | --- | --- | --- |
| Original weight (baseline) | [2.0, 1.5, 1.2, 1.0] | 0.9427 | 0.7639 | 0.8845 | 0.8198 | 0.9779 | 0.7889 |
| Equal-weight baseline | [1.0, 1.0, 1.0, 1.0] | 0.9399 | 0.7542 | 0.8811 | 0.8127 | 0.9768 | 0.7806 |
| Slight perturbation | [2.2, 1.3, 1.0, 1.2] | 0.9405 | 0.7561 | 0.8807 | 0.8136 | 0.9764 | 0.7817 |
| Extreme weight | [3.0, 1.0, 1.0, 1.0] | 0.9428 | 0.7750 | 0.8631 | 0.8166 | 0.9762 | 0.7845 |
| Reverse order weight | [1.0, 1.2, 1.5, 2.0] | 0.9417 | 0.7620 | 0.8781 | 0.8160 | 0.9770 | 0.7843 |

The original weight setting performs optimally on core evaluation indicators, with a significant improvement in performance compared with the equal-weight baseline group. The model performance of the slight perturbation, extreme weight and reverse order weight groups decreases to varying degrees, confirming that the feature weight ranking designed based on biological background is scientific, and differential weighting can effectively improve the model’s chromatin loop recognition ability. Compared with the equal-weight baseline group, the model’s F1-score, AUC and MCC are slightly improved under the original weight, proving that differential weighting of multi-omics features can strengthen the expression of key features and improve model recognition accuracy. All indicators of the slight perturbation group are slightly lower than the original weight, indicating that the original weight is reasonably tuned rather than randomly set. The performance degradation of the extreme weight and reverse order weight groups is more obvious: extreme weight leads to a significant reduction in the Recall indicator, and the overall performance of reverse order weight is also weaker than the original setting. This reversely verifies the rationality of prioritizing higher weights for multi-omics features with stronger correlation with chromatin loop formation, which can effectively avoid recognition bias and overfitting of the model.

**⑤Validation Using Multi-Tool Consensus Hi-C Loops as Gold Standard**

To avoid evaluation bias caused by gold standard selection, the consensus loop results of four mainstream chromatin loop detection tools, Mustache, Peakachu, Chromosight and DloopCaller, were selected as an independent unbiased gold standard, and model performance verification was completed on the chr15, chr16 and chr17 test sets. The key performance indicators are shown as follows:

**Table S5 Performance validated by multi-tool consensus Hi-C loops**

| **Test Chromosome** | **Gold Standard Source** | **Sample Size** | **Accuracy** | **F1-score** | **AUC** | **MCC** | **PR AUC** |
| --- | --- | --- | --- | --- | --- | --- | --- |
| chr15 | Multi-tool consensus Hi-C loops | 748 | 0.9505 | 0.8538 | 0.8377 | 0.9422 | 0.9997 |
| chr16 | Multi-tool consensus Hi-C loops | 590 | 0.9576 | 0.8718 | 0.9996 | 0.8570 | 0.9977 |
| chr17 | Multi-tool consensus Hi-C loops | 562 | 0.9520 | 0.8571 | 0.9999 | 0.8414 | 0.9992 |

The model maintains excellent performance with Accuracy ≥ 0.950, F1 ≥ 0.853, PR AUC ≥ 0.997 on all three chromosomes, and the false negative rate is 0.0000, confirming that the model has no obvious gold standard bias, can effectively identify various chromatin interaction loops based on Hi-C signals, and is not limited to CTCF/H3K27ac-enriched loops, avoiding performance evaluation bias caused by gold standard bias. From chr15 to chr17, there are inherent differences in chromatin structure and loop length distribution among different chromosomes, but the model still maintains stable and consistent high performance, verifying the universal adaptability of the model to different genomic regions. Combined with the verification results of the CTCF/H3K27ac HiChIP functional loop gold standard, the dual gold standard complementary verification can fully ensure the objectivity and comprehensiveness of the model conclusions, proving that the model has stable and reliable recognition performance in both pure Hi-C data and functionally enriched data.

**⑥Cross-Resolution Model Experiments**

TM-Loop is not limited to supporting Hi-C data at 10 kb resolution. The model adopts a parameterized and configurable design, where resolution is an adjustable parameter that does not require modifications to the core network architecture. By simply adjusting configuration items such as resolution and window size, the model can be adapted to Hi-C data at different resolutions. To verify the applicability of the model in multi-resolution scenarios, we completed the adaptation and evaluation test of the model on 5 kb resolution Hi-C data. The key performance indicators are as follows:

**Table S6 Performance at 10 kb and 5 kb Hi-C resolutions**

| **Resolution** | **Accuracy** | **Precision** | **Recall** | **F1-score** | **AUC** | **MCC** | **PR AUC** | **False Positive Rate (FPR)** | **False Negative Rate (FNR)** |
| --- | --- | --- | --- | --- | --- | --- | --- | --- | --- |
| 10 kb | 0.9427 | 0.7639 | 0.8845 | 0.8198 | 0.9779 | 0.7889 | 0.9073 | 0.0473 | 0.1155 |
| 5 kb | 0.9376 | 0.7703 | 0.8241 | 0.7963 | 0.9709 | 0.7601 | 0.8807 | 0.0427 | 0.1759 |

The evaluation results show that the model maintains excellent and stable recognition performance at 5 kb resolution, with all core indicators remaining at high levels. The Accuracy reaches 0.9376 and the AUC is 0.9709, showing no significant decline compared with the baseline performance at 10 kb resolution. The Precision is even slightly higher than that in the 10 kb scenario, indicating that the model can still accurately identify true chromatin loops in higher-resolution data while maintaining a low false positive rate. Although the Recall and F1-score fluctuate slightly due to the increased background noise in higher-resolution data, the overall performance still meets the requirements of the chromatin loop detection task, verifying the good adaptability of the model to Hi-C data at different resolutions. These results demonstrate that TM-Loop has cross-resolution robustness, and can adapt to Hi-C data of different resolutions without modifying the core network architecture. It can be further expanded to adapt to other resolution scenarios according to research needs, providing flexible support for multi-scale chromatin loop detection.

**⑦Zero-shot cross-cell-line generalization test on H1ESC cell line**

To further validate the cross-cell-line generalization capability of TM-Loop across different human biological contexts, we conducted a zero-shot cross-cell-line generalization test using the human embryonic stem cell line H1ESC. Different from the training cell line GM12878 (lymphoblastoid cells), H1ESC represents a pluripotent stem cell context with distinct three-dimensional chromatin organization patterns.

We designed a zero-shot cross-cell-line generalization test where the model was trained exclusively on GM12878 data without any contact with H1ESC data during training. The trained model was then directly applied to H1ESC data for chromatin loop prediction without any parameter fine-tuning or retraining. We selected chromosomes 15, 16, and 17 as the test regions in H1ESC to provide a comprehensive evaluation of cross-cell-line generalization performance.

The quantitative performance metrics of TM-Loop in the GM12878→H1ESC zero-shot cross-cell-line test are summarized in Table S7:

**Table S7 TM-Loop performance in GM12878→H1ESC zero-shot cross-cell-line test (chr15/16/17)**

| **Chromosome** | **Test Samples** | **AUC** | **Recall** | **Precision** | **F1** | **MCC** | **Specificity** | **Calibration Error** |
| --- | --- | --- | --- | --- | --- | --- | --- | --- |
| chr15 | 5502 | 0.9393 | 0.7503 | 0.6200 | 0.6790 | 0.6214 | 0.9207 | 0.0514 |
| chr16 | 4926 | 0.9179 | 0.5815 | 0.5736 | 0.5775 | 0.5041 | 0.9255 | 0.0464 |
| chr17 | 7821 | 0.9405 | 0.7311 | 0.6268 | 0.6749 | 0.6162 | 0.9247 | 0.0441 |

The results show that TM-Loop maintains stable performance when transferred from GM12878 to H1ESC. All three chromosomes maintain AUC values above 0.91, indicating that the model's global discriminative capability shows no significant degradation in the cross-cell-line scenario. The specificity values are all above 0.92, suggesting that the model maintains low false positive rates when facing unseen cellular contexts. Chromosomes 15 and 17 achieve balanced performance with F1-scores of 0.6790 and 0.6749, respectively. Chromosome 16 shows a relatively lower recall (0.5815), which may be attributed to biological heterogeneity between GM12878 and H1ESC cell lines. This minor performance fluctuation reflects normal biological differences rather than model failure, and the overall performance remains at a satisfactory level.

The zero-shot cross-cell-line generalization from GM12878 to H1ESC provides evidence that TM-Loop learns chromatin loop features that are conserved across different human cell types. Combined with our previous cross-chromosome validation in GM12878 and cross-species validation in mESC, we now have a generalization evaluation system covering cross-chromosome, cross-cell-line, and cross-species scenarios. These results demonstrate that TM-Loop possesses certain generalization capability for chromatin loop detection across diverse biological contexts, while broader validation across more human cell types remains an important direction for future work.

**(5)Computational Resource Consumption Analysis**

To evaluate the practical deployment feasibility of TM-Loop, we tested its runtime and memory usage under identical hardware environment, and separately quantified the resource consumption of per-chromosome feature extraction, model training and chromatin loop prediction (data processing + unified inference).

**Table S8 Runtime and maximum memory usage of per-chromosome feature extraction**

| **Chromosome** | **Runtime (s)** | **Peak Memory (MB)** |
| --- | --- | --- |
| chr1 | 8111.93 | 1846.09 |
| chr2 | 6308.46 | 2238.20 |
| chr3 | 5456.33 | 2238.20 |
| chr4 | 3997.91 | 2053.77 |
| chr5 | 4679.58 | 2081.69 |
| chr6 | 5659.40 | 2111.65 |
| chr7 | 4562.22 | 2111.65 |
| chr8 | 3910.97 | 2133.76 |
| chr9 | 3433.18 | 2149.47 |
| chr10 | 4037.98 | 1071.35 |
| chr11 | 5101.45 | 1299.43 |
| chr12 | 4788.93 | 1407.80 |
| chr13 | 1848.87 | 1407.80 |
| chr14 | 3083.47 | 1278.38 |
| chr15 | 2818.20 | 1294.99 |
| chr16 | 2707.92 | 1342.38 |
| chr17 | 4074.09 | 1426.68 |
| chr18 | 1706.86 | 1506.66 |
| chr19 | 2971.83 | 1507.25 |
| chr20 | 2242.36 | 1532.85 |
| chr21 | 753.31 | 1548.66 |
| chr22 | 1629.11 | 1557.89 |
| chrX | 2372.96 | 2071.54 |

**Table S9 Resource consumption of model training**

| **Procedure** | **Total Time (s)** | **Memory Used (MB)** | **Peak Memory (MB)** |
| --- | --- | --- | --- |
| Model Training | 77677.38 | 6780.09 | 8188.90 |

**Table S10 Runtime and memory increment of chromatin loop prediction**

| **Prediction Stage** | **Runtime (s)** | **Memory Increment (MB)** |
| --- | --- | --- |
| Batch 1 (Data Processing) | 47917.25 | 3453.76 |
| Batch 2 (Data Processing) | 49028.02 | 1265.41 |
| Batch 3 (Data Processing) | 48433.57 | 1236.56 |
| Batch 4 (Data Processing) | 49346.03 | 1243.08 |
| Batch 5 (Data Processing) | 50253.45 | 1240.39 |
| Batch 6 (Data Processing) | 50595.40 | 1240.53 |
| Batch 7 (Data Processing) | 51131.06 | 1240.22 |
| Batch 8 (Data Processing) | 48493.63 | 1231.09 |
| Batch 9 (Data Processing) | 50475.66 | 1236.28 |
| Batch 10 (Data Processing) | 49391.88 | 1240.52 |
| Unified Model Prediction | 26264.08 | 53363.84 |

The resource consumption statistics demonstrate that TM-Loop has excellent practical deployment feasibility. Feature extraction presents stable and controllable memory usage across all chromosomes. Model training consumes moderate memory resources, which can run efficiently on standard academic servers. For chromatin loop prediction, the memory increment is progressive and the total resource consumption fits the configuration of conventional high-performance computing clusters. Overall, TM-Loop can be stably applied to chromatin loop detection tasks without relying on extreme computing hardware.

**(6)Change of cluster number of chromatin rings**

Table S11 Change of cluster number of chromatin rings

| **cell/tissue type** | **Chromosome name** | **Prediction loops** | **Clustering parameters（Threshold）** | **Candidate loops** | **Clustered reads** |
| --- | --- | --- | --- | --- | --- |
|  | chr15 | 2953624 |  | 58370 | 591 |
| GM12878 | chr16 | 2707521 | 0.985 | 54322 | 343 |
|  | chr17 | 2766755 |  | 52965 | 689 |
|  | chr15 | 2162914 |  | 33324 | 515 |
| K562 | chr16 | 2080751 | 0.98 | 38515 | 417 |
|  | chr17 | 1977508 |  | 32287 | 670 |
| mESC | chr15 | 2269297 | 0.975 | 42333 | 584 |
|  | chr16 | 2043574 |  | 29045 | 346 |
|  | chr17 | 1915864 |  | 21863 | 378 |

**(7)Number of training sets**

Table S12 Number of training sets

| Chromosome | Positive Samples | Negative Samples | | Chromosome Total Samples |
| --- | --- | --- | --- | --- |
| chr1 | 6862 | | 20586 | 27448 |
| chr2 | 5249 | | 15747 | 20996 |
| chr3 | 4449 | | 13347 | 17796 |
| chr4 | 3195 | | 9585 | 12780 |
| chr5 | 3747 | | 11241 | 14988 |
| chr6 | 4390 | | 13170 | 17560 |
| chr7 | 3529 | | 10587 | 14116 |
| chr8 | 3074 | | 9222 | 12296 |
| chr9 | 2716 | | 8148 | 10864 |
| chr10 | 3190 | | 9570 | 12760 |
| chr11 | 4004 | | 12012 | 16016 |
| chr12 | 3855 | | 11565 | 15420 |
| chr13 | 1552 | | 4656 | 6208 |
| chr14 | 2607 | | 7821 | 10428 |
| chr15 | 2330 | | 6990 | 9320 |
| chr16 | 2262 | | 6786 | 9048 |
| chr17 | 3432 | | 10296 | 13728 |
| chr18 | 1444 | | 4332 | 5776 |
| chr19 | 2580 | | 7740 | 10320 |
| chr20 | 1959 | | 5877 | 7836 |
| chr21 | 657 | | 1971 | 2628 |
| chr22 | 1428 | | 4284 | 5712 |
| chrX | 2059 | | 6177 | 8236 |
| GM12878 Total Samples:282280 | | | | |

**(8) data sources**

Table S13 TM-Loop data sources

| Cell line | Data type | Data source |
| --- | --- | --- |
| GM12878 | Hi-C | https://drive.google.com/file/d/  1rfkdHSfmn5GK7qdzSwVlrSHpJVPPn5R3/  view?usp=sharing |
| K562 | Hi-C | GSE63525 (GEO) |
| mESC | Hi-C | ENCFF289WNN (ENCODE) |
| GM12878 | CTCF ChIA-PET | https://github.com/tariks/peakachu/tree/master/trainingsets |
| GM12878 | Rad21 ChIA-PET |  |
| GM12878 | SMC1 HiChIP |  |
| GM12878 | H3K27ac HiChIP |  |
| K562 | CTCF ChIA-PET |  |
| mESC | SMC1 HiChIP |  |
| GM12878 | CTCF narrowPeak | ENCFF833FTF (ENCODE) |
| K562 | CTCF narrowPeak | ENCFF085HTY (ENCODE) |
| GM12878 | Promoters And enhancers | https://github.com/tariks/peakachu/tree/master/analysis/annotations |
| K562 |  |  |
| mESC |  |  |
| GM12878 | CTCF motif | https://github.com/Yin-Shen/CharID/tree/main/signal |
| GM12878 | ATAC-seq(BigWig) | ENCFF816ZFB (ENCODE) |
| GM12878 | CTCF CHIP-seq(BigWig) | ENCFF797LSC (ENCODE) |
| K562 | ATAC-seq(BigWig) | ENCFF888RNH (ENCODE) |
| K562 | CTCF CHIP-seq(BigWig) | ENCFF910QVN (ENCODE) |
| mESC | ATAC-seq(BigWig) | GSM5588908 (GEO) |
| mESC | CTCF CHIP-seq(BigWig) | GSM723015 (GEO) |
